# Response of Aquatic Bacterial Communities to Hydraulic Fracturing in Northwestern Pennsylvania: A Five-Year Study

**DOI:** 10.1038/s41598-018-23679-7

**Published:** 2018-04-09

**Authors:** Nikea Ulrich, Veronica Kirchner, Rebecca Drucker, Justin R. Wright, Christopher J. McLimans, Terry C. Hazen, Maria F. Campa, Christopher J. Grant, Regina Lamendella

**Affiliations:** 1 Juniata College, Department of Biology, Huntingdon, 16652 USA; 2 Wright Labs LLC, Huntingdon, 16652 USA; 3 University of Tennessee, Department of Civil and Environmental Engineering, Knoxville, 37996 USA; 4 Oak Ridge National Laboratory, Biosciences Division, Oak Ridge, 37831 USA

## Abstract

Horizontal drilling and hydraulic fracturing extraction procedures have become increasingly present in Pennsylvania where the Marcellus Shale play is largely located. The potential for long-term environmental impacts to nearby headwater stream ecosystems and aquatic bacterial assemblages is still incompletely understood. Here, we perform high-throughput sequencing of the 16 S rRNA gene to characterize the bacterial community structure of water, sediment, and other environmental samples (n = 189) from 31 headwater stream sites exhibiting different histories of fracking activity in northwestern Pennsylvania over five years (2012–2016). Stream pH was identified as a main driver of bacterial changes within the streams and fracking activity acted as an environmental selector for certain members at lower taxonomic levels within stream sediment. Methanotrophic and methanogenic bacteria (i.e. Methylocystaceae, Beijerinckiaceae, and *Methanobacterium*) were significantly enriched in sites exhibiting Marcellus shale activity (MSA+) compared to MSA− streams. This study highlighted potential sentinel taxa associated with nascent Marcellus shale activity and some of these taxa remained as stable biomarkers across this five-year study. Identifying the presence and functionality of specific microbial consortia within fracking-impacted streams will provide a clearer understanding of the natural microbial community’s response to fracking and inform *in situ* remediation strategies.

## Introduction

Increasing global reliance on natural gas is a critical issue that has many economic and environmental implications. On average, the global utilization of natural gas exceeds 120 trillion cubic feet (Tcf) per year and is expected to increase at an astounding rate to total 203 Tcf by 2040^1^. In the past decade, technological development has informed many methods of natural gas extraction, as it has become the primary fuel source for energy generation and residential/commercial heating^1,2^. Combinations of horizontal drilling and hydraulic fracturing (fracking) processes have revolutionized the industry by opening up new areas for oil and gas development that were previously inaccessible within the U.S.^3^ The Devonian age (416−359.2 My) Marcellus Shale formation is the largest shale reserve^4^, producing 40% of the U.S. shale gas^5^ and is positioned within the Appalachian Basin, located approximately 1,219–3,000 m below the surface^6^. Production data estimate that as much as 489 Tcf of recoverable resources are contained within the expanse of the Marcellus Shale formation^4,7^.

Briefly, fracking methods involve first drilling vertically, then horizontally, toward the subterraneous gas-bearing formation. Large volumes of fracking fluid, typically composed of water (90%) mixed with sand (9%) and chemical additives (1%), are injected into each well at high pressures to open and enlarge the fractures within the shale formation^8–12^. After the initial fracture process, internal pressure of the rock formation causes fracking fluid in addition to brines, metals, organic compounds, and radionuclides to return to the surface as flowback fluid^3,13,14^. As the well matures, produced water characterized by hydrocarbons, remaining fracking fluid, subsurface brines, and formation solutes is brought to the surface for most of the well life^11,14^.

Research within the last few years has postulated that fracking activity poses critical risks to public health^15–17^ and the environment^18–20^. Fracking practices including treatment of flowback can lead to increased environmental risks for groundwater^21,22^, surface water^20,23,24^, and air pollution^25,26^. In fact, from 2009 to 2015 there were a reported 490 violations connected to improper handling of fracking residual waste and 292 violations for failing to adopt pollution prevention when handling fracking wastewater^27^. Because unconventional methods of natural gas extraction are connected to a reported 30% increase in methane emissions compared to conventional wells, the potential migration of methane into groundwater and the atmosphere is a prominent concern^28^. Alterations in land-use associated with fracking development, mismanagement of fracking fluids, and potential environmental contamination of flowback constituents present possible risks to forested headwater stream ecosystems^29,30^. Small headwater streams are particularly vulnerable to direct pollutant inputs as well as disturbances within nearby riparian terrestrial environments^29^.

While some studies have begun to assess environmental impacts of fracking^31–36^, recent studies have also indicated that robust microbial communities exist within fracking-associated fluids^37,38^. Microbial communities are integral to degrading and metabolizing many of the complex compounds found in the injected and flowback fluids. For example, halotolerant bacteria associated with hydrocarbon oxidation, fermentation, and sulfur-cycling metabolisms including the genera *Halanaerobium*, *Halomonas*, *Vibrio*, *Halolactibacillus*, *Marinobacter*, and autotrophs belonging to *Arcobacter* comprise >90% of the microbial communities within flowback and produced fluids^39^. Moreover, recent studies have indicated that streams within proximity to fracking activities have undergone shifts in their bacterial community structure^35^. For example, Methylocystacea, Acetobacteraceae, *Phenylobacterium*, and Acidobacteriaceae and an increase in methanotrophic bacteria abundance were linked to Marcellus shale activity^40^.

**Figure 1:**
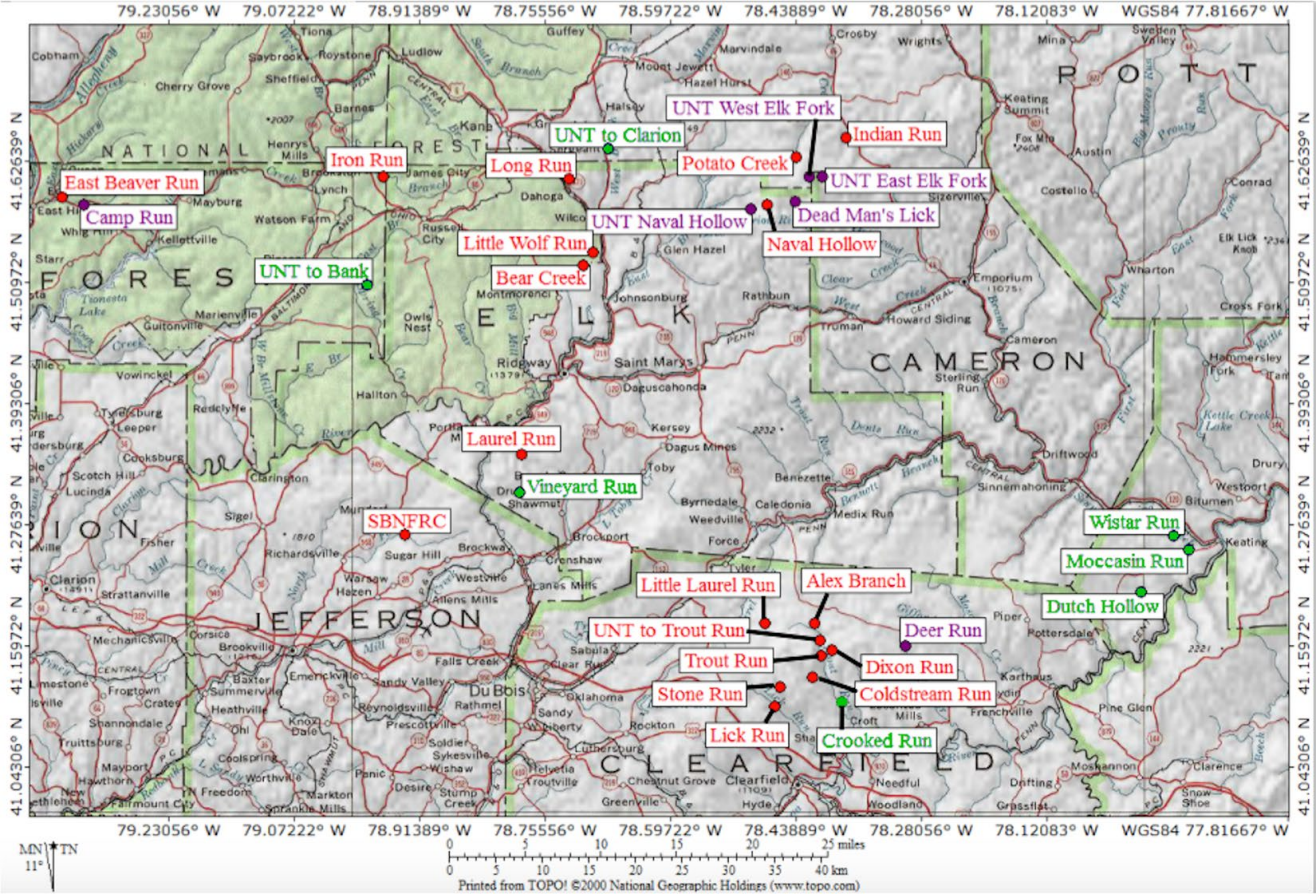
Map of Stream Sites. Stream sites (n = 31) across Clearfield, Jefferson, Forest, Elk, Cameron, and Mckean counties of northwestern Pennsylvania. TOPO! Version 2.6.4 of National Geographic Holdings (https://shop.nationalgeographic.com/category/topo–state-series) was used to generate the map. Red represents MSA+ streams, green represents MSA− and purple represents streams that changed from MSA− to MSA+ during the sampling period (2012–2016). Two streams Findley Run and Diamond Run within southern Blair and Cambria counties not shown on this map are also classified as MSA− streams.

Additional investigations are necessary to monitor aquatic microbial communities and their associated functionality regarding long-term fracking operations. Long-term temporal changes to bacterial structure, water quality, and stream characteristics have yet to be evaluated, especially individual streams that have transformed from pre-fracked to post-fracked. Notably, since 2011, Pennsylvania is second only to Texas in the amount of producing gas wells^41^. This study investigated the bacterial community profiles of 31 headwater stream ecosystems in northwestern PA (Fig. 1) exhibiting different histories of fracking over the course of five years (2012–2016). Streams categorized as having Marcellus Shale activity (MSA+) included streams nearby infrastructure for fracking operations (i.e. wellpads) or active fracking. MSA− streams were not proximal to any associated fracking operations through the duration of the study. For the first time, a temporal investigation of fracking impact on microbial communities within the same watershed was possible with MSA− to MSA+ status changes of six streams. Together, these data permit a powerful and robust identification of biomarker taxa for fracking activity to quantify the broader environmental consequences of fracking operations.

## Results

### Watershed and Stream Measurements

Results indicate that stream pH was significantly different between MSA+ and MSA− streams over all 5 years (median pH: MSA+ = 6.54; MSA− = 7.15; Wilcoxon rank sum test, *p* = *0.001*). Two stream sites, Alex Branch and Little Laurel, which had documented spills prior to sampling, had the lowest median pH levels, 4.96 and 4.5, respectively. Table S1 displays pH, number of active wells, wellpad count, and impact status of each site by year. Stream pH was negatively correlated with number of active wells within the watershed (Spearman’s rho = −0.53, *p* < *0.001*) but no other measures (TDS, salinity, dissolved oxygen, temperature, collection year, and wellpad count) shared a strong relationship with stream pH (Table S3). The number of nearby active wells did not significantly correlate with any other measured parameters (Absolute Value Spearman’s rho = 0.23, *p* < *0.001*). Our previous study identified that watershed land cover (% agriculture, % forested, % wetlands, and forest composition) was not significantly different between these MSA+ and MSA− watersheds in 2013^36^. It is important to note that six streams changed from MSA− to MSA+ during the sampling period and by 2016, only nine streams were classified as MSA− streams (Table 1).

**Table 1:** Stream Classification by Impact Status.

MSA+ Streams (n = 16)	MSA− Streams (n = 9)	MSA− to MSA+ Streams (n = 6)^a^
Alex Branch	Crooked Run	Camp Run (2014)
Bear Creek	Diamond Run	Dead Man’s Lick (2014)
Coldstream Run	Dutch Hollow	Deer Run (2013)
East Beaver Run	Findley Run	UNT to Naval Hollow (2015)
Indian Run	Moccasin Run	UNT East Elk Fork (2015)
Iron Run	UNT to Bank	UNT West Elk Fork (2015)
Laurel Run	UNT to Clarion	
Lick Run	Vineyard Run	
Little Laurel Run	Wistar Run	
Little Wolf Run		
Long Run		
Naval Hollow		
Potato Creek		
Stone Run		
SBNFRC		
Trout Run		

^a^Year of status change is included in parentheses.

### Environmental Drivers of Bacterial Community Composition

**Figure 2:**
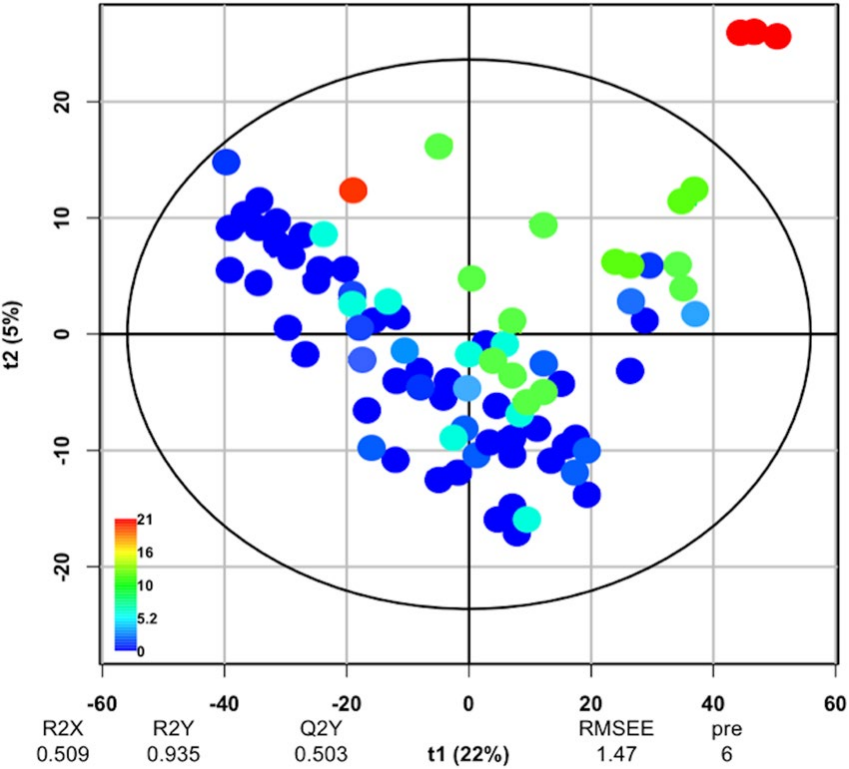
Partial least-squares discriminant analysis (PLS-DA) obtained from sediment samples (n = 86) of both MSA+ and MSA− stream sites. PLS-DA analyses were performed with a CSS normalized OTU table and scores representing each sample are plotted on a PCA plot. Red signifies samples from sites with 21 active wells, and blue signifies zero active wells. The ellipse surrounding the majority of the scores is Hotelling’s T^2^ elliptical tolerance region, which indicated the 95% confidence limits. There is a clear separation of samples between samples with 10–21 active wells and with 0–5 active wells based on the model quality parameters: R^2^X = 0.509, R^2^Y = 0.935, and Q^2^ = 0.503. These significant values represented a cumulative of 6 predictive components calculated by the model and validated by a permutation (n = 10) (p = 0.05).

Partial-least squares linear discriminant analysis (PLS-DA) model revealed that certain environmental parameters could be contributing to microbial community variation within sediment samples (n = 86). Sediment samples from sites with the most active wells (21 active wells) were significantly separated from those with zero active wells, revealing a variation in bacterial communities based on the number of active wells in the watershed (Fig. 2). In the PLS-DA score plot, two axes of variation (t1 and t2) were calculated with the R^2^X, R^2^Y, and Q^2^ parameters of 0.509, 0.935, and 0.0503, respectively (Fig. 2). However, pH (in addition to all other measured water parameters) did not significantly explain differences in the comprehensive microbial composition (sediment, water, bryophyte, biofilm) (Adonis, R^2^ = 0.01, p < *0.022*) or within just sediment samples (Adonis, R^2^ = 0.11, p < *0.0017*). Stream sediments were not found to be statistically different between MSA+ and MSA− status in each year (Table S4).

### Bacterial Community Structure and Diversity

Phylum-level community structure for MSA+ and MSA− samples within each sample matrix revealed Proteobacteria as the dominant phylum (32–71%) across all samples over time. Sediment samples were largely composed of Acidobacteria (6–51%) and biofilm samples ranged in community composition, but Proteobacteria remained the dominant phylum (35–50%) (Fig. S1). Biofilm samples from Little Laurel (LiLR) and South Branch North Fork Redbank Creek (SRC) had high abundances of sequences matching Cyanobacteria (30–40%) (Fig. S2). No major shifts in microbial composition at the phylum level were observed in both MSA+ and MSA− across all sampling years.

Alpha rarefaction curves suggested a reasonable coverage of diversity was reached (Fig. S3). Bryophyte samples were collected only in 2012 and 2013, so they were omitted from downstream temporal analyses. Sediment samples possessed the greatest richness followed by water and biofilm, respectively (Fig. S4). Across all samples, there were no significant correlations between alpha diversity metrics and MSA status (Table S5). Further, for each year, sediments from MSA+ and MSA− sites did not have significantly different richness (Non-parametric two-sample *t*-test, *p* > *0.05)*.

**Figure 3:**
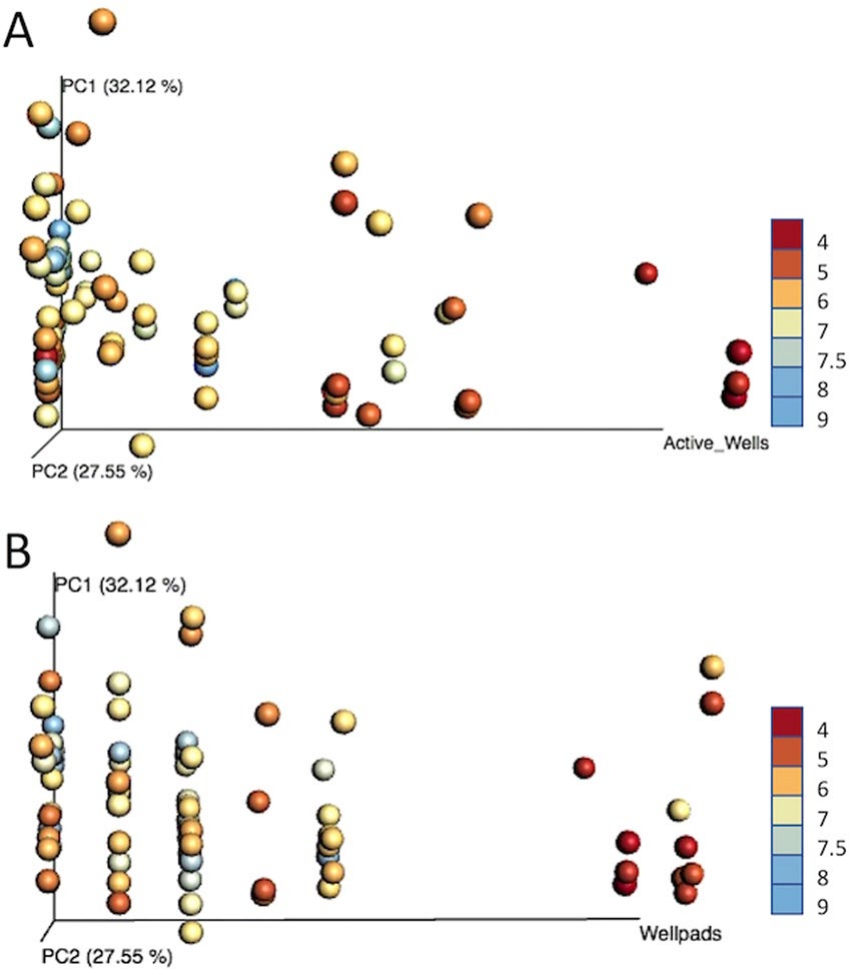
Directional Principal Coordinate Analysis (PCoA) plots of weighted Unifrac distances of sediment samples. The OTU table was CSS normalized and filtered to the genus level. Samples were plotted according to number of active wells (**A**) and wellpads (**B**) along the horizontal axis of the directional PCoA plot. Samples are colored by pH with high pH = blue, low pH = red. The horizontal axis represents from left to right, zero wells and wellpads to 21 active wells and 9 wellpads. Samples are closer in Unifrac distance as the number of active wells increase along PC1, suggesting less variation in bacterial community composition between samples.

Beta diversity of weighted Unifrac distances revealed the possible shaping of bacterial assemblages within members of lower taxonomic ranks. Sample matrix explained the most variation (33.38%) in beta diversity across all samples (Adonis, *p* < *0.001*) (Fig. S5). Further analyses investigated exclusively sediment samples (n = 86), as this matrix had the most comprehensive set of samples across years, and bacteria inhabiting stream sediments act as better proxies for longer term stream impacts^42^. A directional Principal Cooridinates Analysis (PCoA) plot generated using weighted Unifrac distances at the genus level displayed a decrease in variation among samples as the number of active wells (Fig. 3A) and wellpads (Fig. 3B) increased. Samples with zero or few active wells exhibited variable community structure as signified by the spread of samples along PC1 (Unifrac distance: 9.702) (Fig. 3). Samples with many active wells in the watershed were significantly less variable with a Unifrac distance of 0.0569 between the sample cluster and appeared to have greater similarity between samples at the genus level (Fig. 3). Further, a relationship between increased stream water acidity and bacterial community structure as a function of the count of active wells nearby the stream suggested that fracking activity may be selecting for specific bacterial assemblages within impacted stream environments.

### Identification of Microbial Indicators

**Figure 4:**
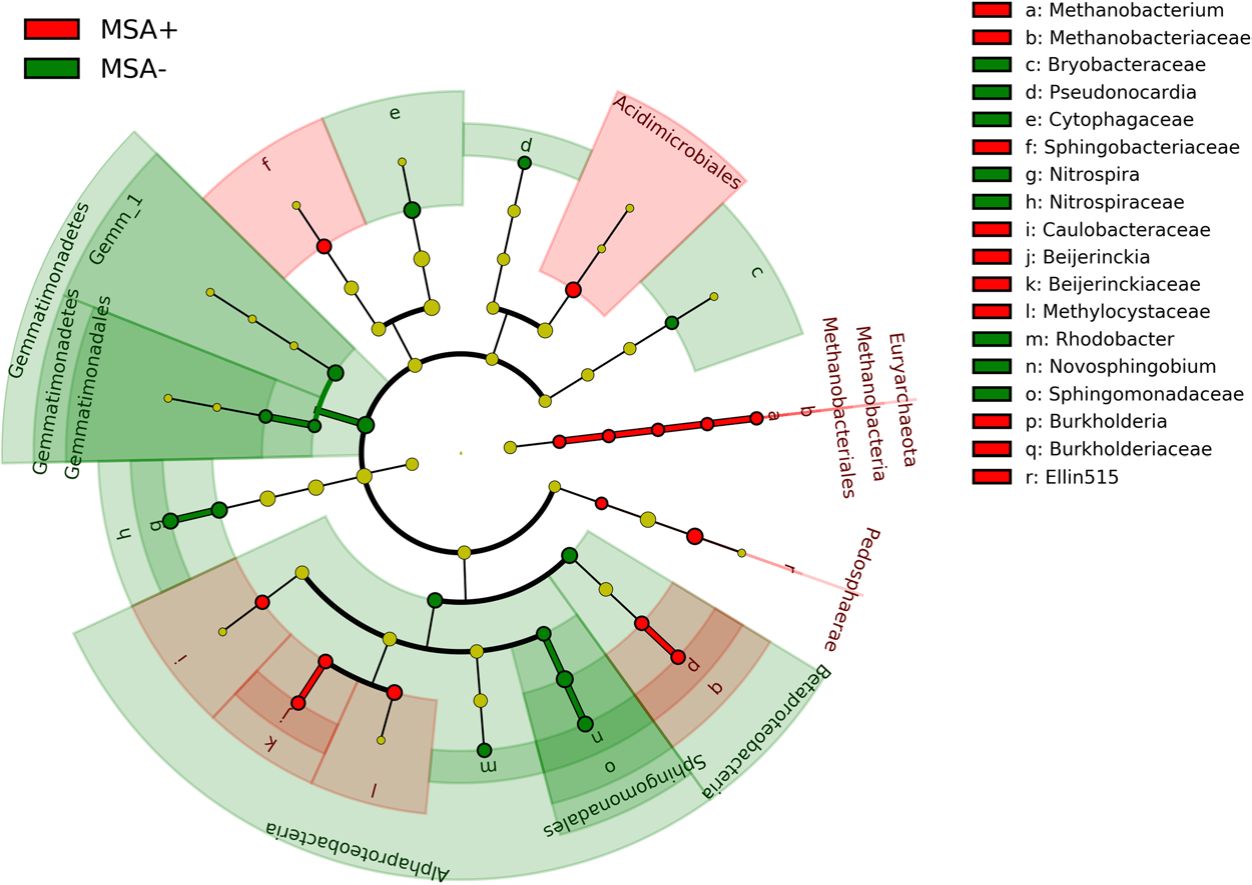
LEfSe plot for clades of bacteria enriched within MSA+ and MSA− streams. The cladogram reports the taxa (highlighted by small circles and shading (MSA+ = red; MSA− = green) that are enriched within corresponding sediment samples. LEfSe utilizes Kruskal-Wallis to determine significantly different taxonomic features (p < 0.01) between experimental groups, a pairwise Wilcoxon rank sum statistic to test biological consistency across subgroups (p < 0.01), and finally a linear discriminant analysis (LDA score >2.0) to determine the effect size, or magnitude of variation of the features between groups.

Biomarker analyses revealed an enrichment of specific OTUs within MSA+ and MSA− sediments, suggesting that certain OTUs could be readily responding to environmental perturbations (Fig. 4). In MSA− streams, members of Gemmatimonadetes were >3 log_10_-fold more abundant and *Myxococcus*, *Rhodobacter*, and Sphingomonadales members were >2 log_10_-fold more abundant. Methanobacteriaceae, Methylocystaceae, Beijerinckiaceae, Caulobacteraceae, and members of Pedosparaea were amongst the most significantly enriched in MSA+ sediment samples (Fig. 4). Specifically, both *Methanobacterium* and *Beijerinckia* were >2 log_10_-fold more abundant in MSA+ streams. Specific taxa were consistently enriched across many sampling years when biomarker analyses were completed with OTU relative abundances filtered by year to account for possible pseudoreplication (Fig. S6). For example, *Methanobacterium* sequences were enriched in MSA+ sites from 2012–2014 while Beijerinckiaceae sequences were increasingly present in later samples (2014–2015). Members of Verrucomicrobia, Sphingomonadales, and Myxococcaceae were consistently enriched in MSA− sites each year. Further, many of these bacteria also shared strong positive relationships with increased number of wellpads within the watershed, including taxa within the Caulobacteraceae, Methanobacteriaceae, Acetobacteraceae, and Methylocystaceae families (FDR-adjusted p < 0.05).

**Figure 5:**
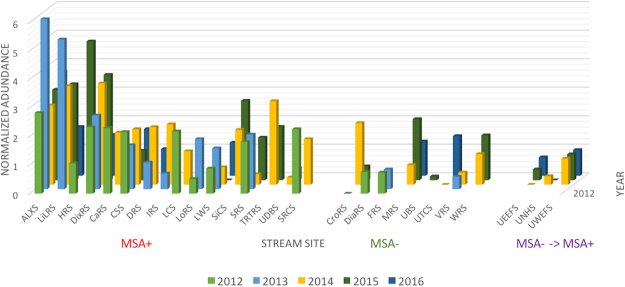
Relative abundance plot of *Methanobacterium* sequences in sediment samples (n = 86). Samples were grouped by MSA+, MSA−, and MSA− to MSA+ status. A filtered OTU table that underwent CSS normalization was used, showing stream site along the x-axis and year along the z-axis. *Methanobacterium* presence was stable across years and showed greater abundance in MSA+ sites.

A survey of *Methanobacterium* abundance in sediment samples across all stream sites confirmed that the enrichment of these archaeal taxa was not singular to one stream site. Several MSA+ sites including both documented spill sites Alex Branch (ALXS) and Little Laurel Run (LiLRS) had the distinctly higher abundances of *Methanobacterium* each year (Fig. 5). *Methanobacterium* accounted for 0.14% of sequences in Alex Branch and 0.13% in Little Laurel in 2013. Indian Run (HRS) also exhibited significantly higher abundance of *Methanobacterium* in 2015 (0.11%), during which there were a total of 12 active wells within the watershed. Many other stream sites consistently harbored *Methanobacterium*, both within MSA+ and MSA− sites; however, normalized abundances did not exceed 3 for MSA− sites (Fig. 5).

The apparent enrichment of different OTUs across all years between MSA+ and MSA− sites led us to investigate whether MSA status could be predicted by differential OTU abundance. PLS-DA analyses showed that active well count was the most significant measured environmental selector, which directly corresponded to active fracking. Therefore, samples were reassigned as fracked (HF+) and non-fracked (HF−) based on actively producing wells (rather than wellpad presence). A random forest model was performed with an OTU table combined with measured environmental data, revealing that active wells, stream, wellpads, and pH were all significant predictors of fracking status. Random forest modelling was continued with only the OTU table to identify potential microbial predictors of active fracking activity based on differences in OTU abundances. Thirty OTUs with the largest mean decrease in Gini index were selected (Table S6). The three most significant predictors were Sphingobacteriales, Cytophagaceae, and Solibacterales, signifying they had a large difference in their abundances between HF+ and HF− sites and could consequently represent accurate predictors fracking status for a site. Of the top thirty predictors identified by random forest, three were significantly enriched in MSA+ streams and twelve were significantly enriched in MSA− streams. The percentage classified correctly value for all 100 random forest models was calculated to be 0.686, indicating that the random forest models predicted fracking status at a percentage higher than expected by chance.

**Figure 6:**
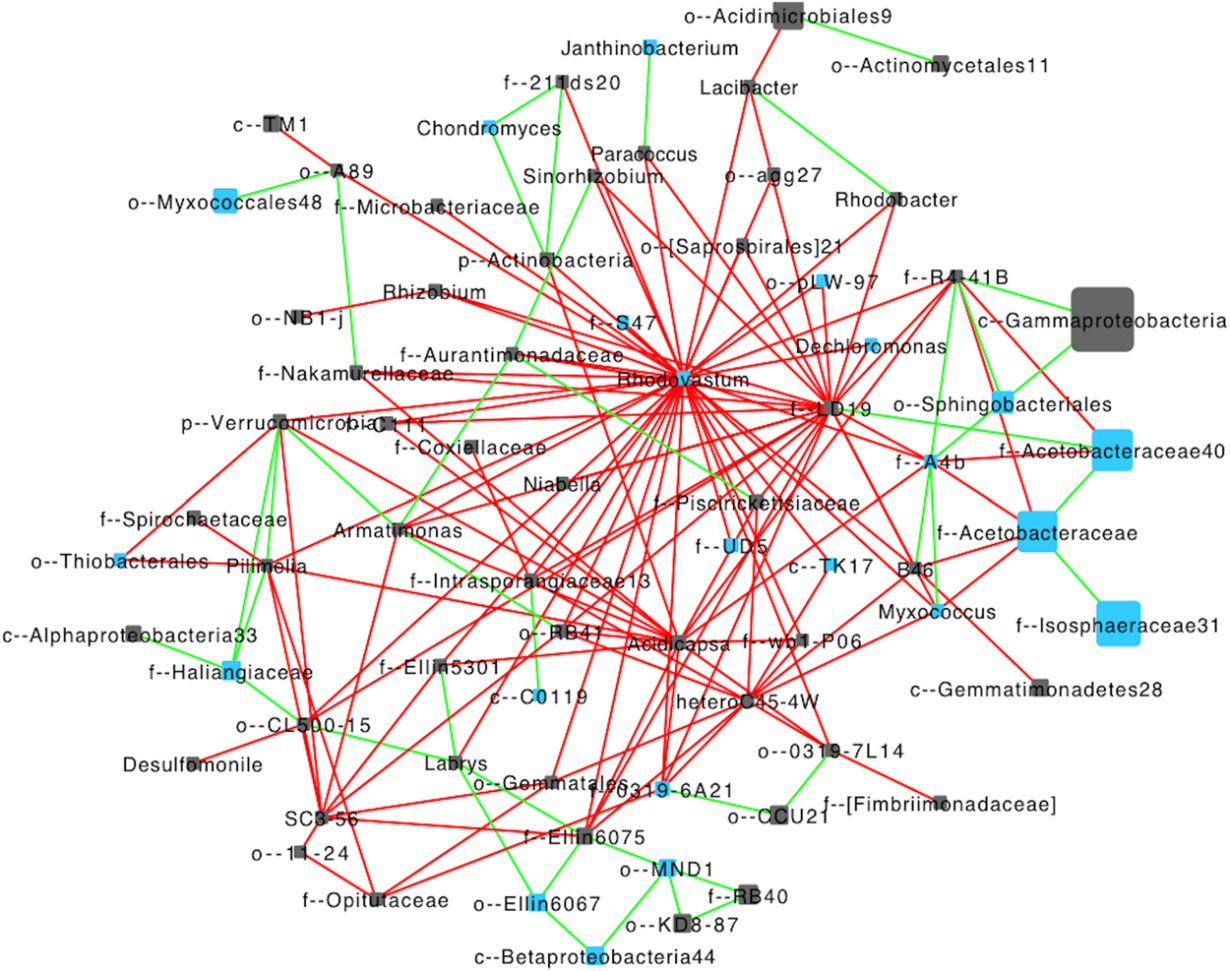
Co-occurrence network bacterial taxa within a selection of MSA+ (n = 14) stream sites. The network plot was generated within the Cytoscape plug-in Conet and reveals strong positive (Spearman’s rho >0.8) and strong negative (Spearman’s rho <−0.8) correlations. Edges connecting nodes highlighted in green are indicative of strong positive correlation, whereas edges highlighted in red are indicative of a strong negative correlation. Sizes of bacterial nodes indicate bacterial abundance. Taxa identified as important predictors are colored in blue.

Co-occurrence network patterns of bacterial communities within both MSA− and MSA+ stream sediments displayed strong relationships. A subnetwork containing bacterial correlations within MSA+ streams revealed that there were strong positive and negative correlations between taxa that were previously identified as important predictors of nearby fracking activity (Fig. 6). For example, a positive relationship was revealed between several OTUs within the Acetobacteraceae family. Further, Acetobacteraceae shared the only positive correlation with the family LD19 within the Methylacidiphales order, which had 23 negative correlations with other OTUs. Gammaproteobacteria were the most abundant group, and shared positive correlations with taxa of the Sphingobacteriales order and family R4–41B within the Pedosphaerales order (Fig. 6). Sequences matching to *Rhodovastum*, a member of the Acetobacteraceae family, shared the most negative correlations (37) with other OTUs.

## Discussion

Five years of stream water chemistry data indicated that stream sites located in watersheds with Marcellus Shale activity (MSA+) had lower pH than MSA− sites. While these differences could be partly attributed to a number of variants (i.e. watershed characteristics, disparities in acid rain deposition, or fracking activity), sites were selected using GIS surveys to avoid potential confounds^36^. Further, it has been shown that pH significantly differs in sites with and without fracking activity^36,40,43,44^. Observed differences in pH of stream water could be attributed to weathering of pyritic geological formations^45^ exposed during the drilling process or exposure to concentrated acids that are used within fracking fluids during the hydraulic fracturing process^33,40^. A negative correlation between the number of active wells and pH suggests that activity associated with actively producing wells could be increasing the acidity of stream ecosystems. Indeed, streams with the largest number of active wells, including the two documented spill sites, had significantly lower pH levels. It is important to note that stream sites selection excluded streams impacted from acid mine drainage. No other measured water chemistry measurements were significantly different, suggesting that pH differences were not related to differences in the sites that were selected but rather the effects of activity.

There are a few modes in which fracking could be shaping bacterial community dynamics. Numerous studies have previously documented apparent shifts in bacterial community resulting from changes in stream water pH^46–48^. Fracking activity could be causing differences in stream pH and thereby causing shifts of ecosystem dynamics^49^ and communities^40,43,44^. It is also possible that aquatic bacteria are responding directly to fracking inputs. While alpha diversity was not indicative of extensive trends between MSA+ and MSA− streams (Table S5), the potential inputs from fracking-associated fluids during active fracking may not be frequent enough or encompass a large enough volume to cause a drastic restructuring of the aquatic bacterial communities. It is clear, however, that spills associated with fluid mishandling and other operational accidents immediately impacted nearby surface waters^50^. While older and inactive wells remain a critical concern for environmental contamination associated with equipment failure^51^, operating wells are associated with increased truck transportation and fluid relocation, which could pose larger potential threats to the surrounding environment. PA treats most of its fracking wastewater off site, requiring extensive relocation of fluids following the fracking process^33^. Injection and flowback fluids are documented to have pH range of 6–8, but the concentration of acids (and bases) varies widely by company and well site^52^.

Significant differences in beta diversity were observed at the family and genus-level (Fig. 3). As active wells increased, Unifrac distances between samples decreased, suggesting a shaping of the sediment bacterial communities (Fig. 3). Samples with zero active wells exhibited large variation in comparison to samples with as many as 21 active wells. This disparity in bacterial variation could be resulting from differential enrichment of bacterial assemblages that vary in their sensitivity to changes in stream conditions^53^. Previous studies investigating fracking impact on nearby aquatic systems have reported increased methanotrophic bacteria in impacted sites^31,40^. Specifically, in our study, members of the Caulobacteraceae, Beijerinckiaceae, Methylosystaceae, and Burkolderiaceeae were significantly enriched in MSA+ sites (Fig. 4). Caulobacteraceae, Beijerinckiaceae, and Methylosystaceae are known to contain bacteria with methanotrophic capabilities^54^. Further, Beijerinckiaceae and Methylocystaceae are also commonly found in acidic habitats, and they often prevail in methane-emitting wetlands^55,56^. Both Caulobacteraceae and Burkolderiaceae are composed of taxa with great metabolic versatility, as they can degrade a wide range of hydrocarbons^57^.

Interestingly, among the bacteria most enriched in MSA+ stream sites were *Phenylobacterium*, which may be capable of degrading phenyl-compounds and other complex hydrocarbons in acidic environments^58,59^. Random forest analysis revealed that *Phenylobacterium* as well as many of the enriched biomarkers were important predictors of fracking activity. Moreover, *Rhodovastum*, a member of the Acetobacteraceae family, appeared to be a hallmark of MSA+ stream sites, indicated by its high degree of negative associations with other taxa (Fig. 6). *Rhodovastum* has not been extensively studied but has been isolated from methane-rich paddy soils^60^ and is a putative hydrocarbon-degrader within methane-emitting fen soil^61^. The large degree of biological interactions within MSA+ sediment soil suggests that *Rhodovastum* could be among those participating in degrading hydrocarbon constituents of potential fracking inputs. Bacterial genera known to degrade aliphatic and aromatic compounds have been previously identified in fracking-associated fluids^37,39,62^.

Enrichments in MSA− sites were comprised of several pH sensitive taxa, which were potentially repressed in MSA+ stream conditions. Gemmatimonadales favor neutral pH and have been found to decrease in abundance in more acidic conditions^63^. Additionally, Nitrospira is found in greater abundance in higher pH within soils^47^, which is consistent with their enrichment in MSA− sites. Furthermore, certain biomarker taxa remained significantly enriched across individual sampling years. From 2012 to 2015, these biomarker taxa were repeatedly observed at significantly higher abundance when fracking activity was most prevalent across northwestern PA. In 2016, the total number of active wells across sample sites dropped to 37 from 116 in the previous year. Samples in 2016 contained different bacterial enrichments compared with previous years’ (Fig. S6).

16 S rRNA gene sequencing revealed that methanogenic taxa were significantly enriched in MSA+ sites during the five-year period. Sequences belonging to archaeal taxa were more abundant in MSA+ sites, including spill sites, and were dominated *Methanobacterium* (Fig. 5). This finding is consistent with a study that found downstream sites of unconventional oil and gas wastewater releases were dominated by *Methanomicrobia*, also methanogenic bacteria, which was attributed to changes in stream geochemistry following fracking wastewater inputs^31^. These observed differences are characteristic of unaerated and biocide-amended impoundments of produced water from unconventional oil and gas drilling^64^, suggesting that MSA+ streams may share similar bacterial assemblages as those that have endured fracking-related spills. Methanogens are tolerant of acidic conditions, enabling them to survive in such environments^65^.

The concomitant enrichment of both methanogenic and methanotrophic bacteria suggests that they may be co-occurring due to stream conditions. The increased acidity in MSA+ streams has potentially enriched methanogens^66^, and the greater availability of both biogenic methane and potential methane emission from fracking has cultivated an enrichment of methanotrophic bacteria. Methanogenic and methanotropic bacteria have been shown to coexist in sediment habitats^67^, but it remains inconclusive whether methane contamination is frequent enough nearby fracking activity to amplify this relationship. Stream sediments naturally contain methane and its intermediate constituents, and therefore, harbor methanogenic archaea, essential for methane production and consumption within the freshwater ecosystem^68,69^. However, subsets of groundwater wells <1 km from shale gas wells in PA have documented elevated concentrations of methane, ethane, propane, and methane isotopic signatures consistent with a thermogenic source, suggesting potential methane migration from fracking^70–72^. Conversely, it is reported that groundwater in northwestern, VA was not contaminated from the installation and fracking of shale-gas wells over the course of three years^50^.

While high-throughput sequencing of the 16 S rRNA gene enabled us to identify bacteria responsive to proximate fracking activity, this approach should be employed with caution, due to the limited phylogenetic resolution when using the 16 S rRNA gene as a target. Future work such as shotgun metagenomics and metatranscriptomics could be used to investigate the functional response of microbial communities towards potential environmental disturbances associated with Marcellus shale activities. Future work should also focus on additional chemical measurements within stream water such as methane concentration and isotopic carbon to further connect abiotic conditions to microbial response, a current limit of this study. Future studies will expand to other areas of PA with greater presence and documented impacts Marcellus shale activity to investigate the spatial stability of in-stream biomarker taxa identified in this study.

Altogether, this study highlighted stable bacterial taxa responding to Marcellus shale activity and further supplements a longitudinal correlation of increased acidity of stream water and fracking activity adjacent to headwater streams over five years. While overall bacterial community composition did not show large-scale differences between MSA+ and MSA− streams, our results suggest that fracking activity may still be shaping community dynamics of select bacterial assemblages. These findings are relevant, as small headwater streams may be most impacted by the disruption associated with fracking operations, and these *in situ* bacterial communities comprise the first biological response. Understanding the dynamics of these aquatic bacterial communities and their potential capabilities will assist in attenuation of impacted sites and further inform environmental agendas associated with fracking operations.

## Methods

### Site Selection

All streams selected for sampling were located on public lands with necessary permits acquired through the Department of Conservation and Natural Resources (http://www.dcnr.state.pa.us) and the Pennsylvania Game Commission, SFRA-1322. All permits are available upon request.

Thirty-one Pennsylvanian headwater streams with unconventional shale gas well permits from the PA DEP were selected based on previously outlined criteria^44^. The selected streams were remotely located within the Marcellus shale region in northwestern PA within forested watersheds that had little to no prior anthropogenic activity. Further, the streams contained naturally reproducing wild brook trout populations. All sites shared similar watershed and stream characteristics to allow comparison with respect to the effect of Marcellus shale activity and fracking^44^. Sampling locations are displayed in Fig. 1.

Streams without fracking infrastructure development for the duration of the sampling period (2012–2016) were classified as lacking Marcellus shale activity or MSA− (n = 9). It is important to note that Dutch Hollow within the MSA− grouping had land cleared and roads constructed prior to sampling. Streams with at least one wellpad were categorized as MSA+ (n = 19). A wellpad was defined as land cleared for drilling operations that is occupied by a minimum of one well. Well presence on a wellpad was confirmed by the spud date or the date on which the ground was penetrated to drill the well. Because Alex Branch and UNT to Trout Run were both MSA+ streams and the sole inputs to Trout Run, Trout Run was categorized as MSA+ as well. Six streams changed from MSA− to MSA+ between 2012–2016 and were grouped separately. Two MSA+ streams (Little Laurel Run and Alex Branch) had documented fracking-associated contamination within the watershed before sampling began in 2012 according to the PADEP. For additional analyses, stream sites were also categorized as fracked (HF+) and non-fracked (HF−) based on actively producing wells within the watershed. Active wells within a watershed were defined as operating wells that were producing fluids for natural gas extraction.

Detailed information of selected watershed characteristics and impact status of each site over 5 years can be found in Supplemental Information (Table S1). It should be noted that not all sites were sampled every year because of high flow and/or inaccessibility.

### Field Sampling

Sediment, water, bryophyte, and biofilm samples were collected over 5 years (2012–2016) in the summer months of June and July. Methods for sterile sample collection were utilized as described in Trexler *et al*. (2014). Sediment samples (n = 86) were collected using sterile scoops from areas adjacent to the water-bank interface. Biofilm samples (n = 5) were collected in sterile 50 mL conical tubes. Bryophyte samples (n = 20) were cut directly from submerged rock substrates with a sterile scalpel and consisted of two common water mosses, *Fontinalis sphagnifolia* and *Fontinalis antipyretica*. It should be noted that bryophyte samples were only collected in 2012 and 2013. Water samples (n = 78) were sampled by collecting 1 liter of water in a sterile Nalgene bottle from a central riffle. Water samples were filtered on site with 0.22 μm polyethersulfone filters (Millipore, Billerica, MA) and stored in sterile Whirl-Pak bags (Nasco, Fort Atkinson, WI). All samples were placed immediately on ice and stored at −80 °C. Stream water chemistry measurements including: temperature, pH, conductivity, salinity, and total dissolved solids (TDS) were taken on site at the time of sampling with a weekly-calibrated PCSTestr 35 (Oakton Instruments, Vernon Hills, IL).

*DNA Extraction and 16 S rRNA library preparation*. Nucleic acid extractions were performed on water filters, sediment, bryophyte, and biofilm samples using a modified cetyltrimethylammonium bromide (CTAB) phenol-chloroform-isoamyl alcohol method, as described by Hazen *et al*.^73^. The pellet was resuspended in 30 μL buffer EB (Qiagen, Germantown, MD) and the DNA was then subsequently subjected to the AllPrep DNA/RNA Mini Kit (Qiagen, Germantown, MD), using the manufacturer’s suggested protocol. The DNA extracts were quantified using the Qubit 2.0 fluorometer double-stranded DNA (dsDNA) high sensitivity DNA kit (Invitrogen, Carlsbad, CA) according to the manufacturer’s instructions and stored at −20 °C.

Duplicate 25 μL Illumina tag Polymerase Chain Reactions (PCR) from each sample (n = 281) contained final concentrations of 1× PCR buffer, 0.8 mM dinucleoside triphosphates (dNTPs), 0.625 U of *Taq* polymerase, 0.4 μM 515 F forward primer, 0.4 μM Illumina 806 R reverse barcoded primer, and ~10 ng of template DNA per reaction. Sediment DNA extracts, in many cases, were diluted by 1:10 in DEPC-treated water to achieve successful amplification. PCR was performed on an MJ Research PTC-200 thermocycler (Bio-Rad, Hercules, CA) using cycling conditions of 94 °C for 3 min, followed 35 cycles of 94 °C for 45 s, 53 °C for 60 s, and 72 °C for 90 s, and ending with 72 °C for 10 min. PCR reactions were kept at 4 °C until visualized on a 2% agarose E-gel (Invitrogen, Carlsbad, CA) stained with ethidium bromide. Pooled PCR products were gel purified using the Qiagen Gel Purification Kit (Qiagen, Frederick, MD), quantified using the Qubit 2.0 Fluorometer (Life Technologies, Carlsbad, CA), and validated using the Agilent Bioanalyzer High Sensitivity DNA kit (Agilent Technologies, Santa Clara, CA, USA) prior to submission for sequencing.

### Sequencing

Due to the temporal nature of this study, libraries were sequenced by different facilities depending on the year. 2012 samples were sequenced using Illumina MiSeq set for 250 bp paired-end chemistry^40^. 2013 samples were sequenced through the EMP Consortium on the Illumina HiSeq. 2000 platform using the single-end 100 bp chemistry. Finally, libraries from 2014–2016 were sequenced on the Illumina Miseq platform with either the 150 bp or 250 bp paired-end chemistries. To normalize for different sequencing runs, we only used the first 100 bp of the forward reads. Quality control stringency and same-length truncation produced no significant run-to-run variation. Libraries from 2012 to 2016 were compiled into one multiplexed file for downstream sequence analysis. Sequence data for this project were deposited in the NCBI Sequence Read Archive under accession number SRP114850 (http://www.ncbi.nlm.nih.gov/sra).

### Bioinformatics and Statistical Analyses

Sequence reads were trimmed at a length of 100 base pairs to normalize for different sequence lengths and quality filtered at an expected error of less 0.5% using USEARCH v7^74^. After quality filtering, the reads were analyzed using QIIME 1.9.0^75^. Open reference operational taxonomic units (OTUs) were picked and chimeric sequences were identified using USEARCH7^76^. OTU taxonomy was assigned using Greengenes 16 S rRNA database (13–5 release, 97%)^77^. The OTU table was filtered further to discard samples with less than 5400 sequences to remove samples with low sequencing depth because some diversity estimators can be sensitive to varying sample sizes. An additional filtering step was performed to discard OTUs that represented less the 0.005% sequences as recommended for Illumina-generated sequence data^78^. A total of 18.1 million sequences represented 189 samples (see Table S2 for filtering information for each sample).

Alpha-diversity multiple rarefactions were conducted using QIIME 1.9.0 on sequences across all samples from all years (2012–2016). To generate alpha diversity rarefaction curves multiple rarefactions were conducted on sequences across all samples from minimum depth of 500 sequences, to a maximum depth of 10,000 sequences, with a step size of 500 sequences/sample for 10 iterations. Alpha rarefactions were then collated and plotted using Chao1 and observed species richness metrics. Alpha diversity comparisons between sample matrix, stream status (MSA+ or MSA−), and measured water chemistry were conducted using two-sample t-test and nonparametric Monte Carlo permutations (n = 999). Visualization of trends in microbial community structure for MSA+ and MSA− samples were generated in R using the *phyloseq* package version 1.12.2^79^.

Partial least square discriminant analysis (PLS-DA) was utilized to predict differences in sediments from streams with different numbers of proximal active wells. A cumulative sum scaling (CSS) normalized, unrarefied OTU table containing only sediment samples was used to create an OTU count matrix (X) and active well counts (Y) were used to predict maximal covariance of the samples. A six-component model was generated and validated by permutation (n = 10) (p = 0.05) to explain the relationship between the inputs (X variables) and the outputs (Y variables). Model quality was assessed by cross validation parameters R^2^X, R^2^Y, and Q^2^. Cumulative R^2^X and R^2^Y values represent fraction of variance of the X and Y matrix while Q^2^ represents the predictive accuracy of the model. A normalized OTU table was used to perform beta diversity analysis. Beta diversity was calculated using the weighted Unifrac distance metric and principal coordinate analysis (PCoA) plots were visualized using EMPeror^80^. Mean Unifrac distances were calculated between sample clusters were reported. Adonis tests were performed on the weighted Unifrac values to determine the variation explained by stream water characteristics, sample year, and watershed characteristics. All statistical analyses were considered significant at α = 0.05 for both continuous and categorical variables.

Linear discriminant analysis (LDA) effect size (LEfSe) was utilized to identify specific bacterial taxonomic biomarkers unique to MSA− and MSA+ communities within sediment bacterial communities. The LEfSe method couples tests for statistical significance with other tests of effect relevance and biological consistency to determine the features, in this case OTUs, that most likely explain the differences in a phenotype or condition^81^. A normalized OTU table filtered to the genus-level and properly formatted^81^. OTU comparisons were performed with “impact status” (MSA+ or MSA−) set as the main categorical variable and “sample matrix” as the secondary categorical variable. Alpha levels of 0.01 were used for both the Kruskal-Wallis and pairwise Wilcoxon tests. Significant features with LDA >2 were plotted. The ‘plot_cladogram’ function was used to generate a cladogram for visualization of relationships between enriched bacterial taxa. To address the possibility of pseudoreplication in biomarker analyses, LEfSe was performed separately for each respective year (Fig. S6).

Statistical analysis of watershed characteristics (stream water pH, total dissolved solids (TDS), salinity, and temperature) as well as the number of active wells and wellpads were conducted between MSA+ and MSA− streams using Wilcoxon rank sum test in R. Data was transformed (log_10_) and statistical significance was considered at α = 0.05. Spearman correlations were calculated to examine the relationship between continuous abiotic variables of the stream sites.

### Random forest modeling

The *randomForest* package in R^82^ was implemented to produce a model that predicted fracking status of sediment samples using OTUs as predictors. The dataset consisted of un-normalized OTU counts with samples reclassified as fracked (HF+) or non-fracked (HF−). The initial step in the model generation was the separation of the full dataset into a training set and a test set, of which the training set consisted of a randomly selected subset containing approximately 80% of the samples and the test set consisted of the remaining 20% of the samples. Because no repeated samples existed within the dataset, the *sample* function was used to generate the randomly selected subset. The *randomForest* function was run on the training set, with the *mtry* parameter set to the square-root of the total number of predictors and all other default settings. To test the model, the *predict* function was run on the random forest model generated for the training set to predict the fracking status of the samples within the test set. The estimated test error was calculated as the total number of correct predictions divided by the total number of predictions made. The most important predictors were identified by implementing the *importance* function on the random forest model. The Mean Decrease in Gini Index was used as the measure of variable importance. The random forest model generation process was repeated 100 times so that the random forest model was run on 100 randomly selected training sets. An average estimated test error was calculated for all repetitions of the process. The top 30 predictors were assigned using the calculated average Mean Decrease in Gini Index of the 100 random forests produced.

### Co-occurrence networks

Co-occurrence network plots generated in Cytoscape with the CoNet plug-in were used to investigate relationships between OTUs within MSA+ streams. A selection of 14 samples in streams representing all years (2012–2016) were used within the analysis. OTUs appearing in less than seven samples were discarded from the CSS normalized OTU table. Spearman correlation, Bray-Curtis dissimilarity and Kullback-Leibler dissimilarity were used to select edges. Thresholds for edge selection were chosen by CoNet such that the initial network would contain the 150 edges with the most positive correlations and the 150 edges with the most negative correlations for each of the four methods. The networks were then refined with a row-shuffling permutation step with 100 iterations. After permutation, the samples were renormalized. The permutation-renormalization step was intended to mitigate the compositionality of the data as described in Faust *et al*.^83^. The networks were further refined with a bootstrap step with 100 iterations. The networks created in the initial, permutation-renormalization, and bootstrap steps were combined using the Brown p-value merge and Benjamini-Hochberg multiple testing correction. The threshold for merged p-values was set at 0.05. Spearman rho thresholds were −0.82 and 0.85; thresholds of Bray-Curtis distances were 0.10 and 0.78.

## Electronic supplementary material

Supplementary Information
