# Supplementary material for: Response of Aquatic Bacterial Communities to Hydraulic Fracturing in Northwestern Pennsylvania: A Five-Year Study
Source: Sci Rep. 2018 Apr 9;8:5683. doi: 10.1038/s41598-018-23679-7 (PMC5890257; doi:10.1038/s41598-018-23679-7)
Supplement: Supplementary file 1 — Supplementary Information [file 41598_2018_23679_MOESM1_ESM.pdf]

Supplemental Information

Response of Aquatic Bacterial Communities to Hydraulic Fracturing in Northwestern Pennsylvania: A Five-Year Study

Nikea Ulrich[1], Veronica Kirchner[1], Rebecca Drucker[1], Justin Wright[2], Christopher McLimans[1], Terry Hazen[3,4], Maria F. Campa[3,4], Christopher Grant[1], Regina Lamendella[1,2*]

[1]Juniata College, Department of Biology, Huntingdon, 16652, USA
[2]Wright Labs LLC., Huntingdon, 16652, USA
[3]University of Tennessee, Department of Civil and Environmental Engineering, Knoxville, 37996, USA
[4] Oak Ridge National Laboratory, Biosciences Division, Oak Ridge, 37831, USA
[*]lamendella@juniata.edu

Table S1. Stream sites selected parameters: pH, number of active wells and wellpads, and impact status by year

Status: MSA+

**Stream**		**2012**	**2013**	**2014**	**2015**	**2016**
ALXS	Impact Status	MSA+	MSA+	MSA+	MSA+	MSA+
	Active Wells	9	0	9	9	9
	Wellpads	9	9	9	9	9
	pH	4.88	4.57	4.96	4.98	5.1
SiCS	Impact Status	MSA+	MSA+	MSA+	MSA+	MSA+
	Active Wells	2	2	2	2	2
	Wellpads	1	1	1	1	1
	pH	6.12	5.1	5.6	6.13	5.71
CaRS	Impact Status	MSA-	MSA-	MSA+	x	x
	Active Wells	0	0	0	x	x
	Wellpads	0	0	0	x	x
	pH	7.4	7.75	7.28	x	x
CSS	Impact Status	MSA+	MSA+	MSA+	MSA+	MSA+
	Active Wells	5	5	5	5	5
	Wellpads	4	4	4	4	4
	pH	6.18	6.67	6.19	8.26	6.3
DixRS	Impact Status	MSA+	MSA+	MSA+	MSA+	MSA+
	Active Wells	0	0	0	0	0
	Wellpads	2	2	2	2	2
	pH	7.61	5.8	6.16	5.71	7.01
EBRS	Impact Status	MSA+	MSA+	x	x	x
	Active Wells	0	0	x	x	x
	Wellpads	2	2	x	x	x
	pH	7.99	7.62	x	x	x
HRS	Impact Status	MSA+	MSA+	MSA+	MSA+	x
	Active Wells	6	6	6	13	x
	Wellpads	2	2	2	2	x
	pH	7.2	6.87	6.46	6.76	x
IRS	Impact Status	MSA+	MSA+	MSA+	x	x
	Active Wells	0	0	0	x	x
	Wellpads	1	1	1	x	x
	pH	6.94	6.16	6.98	x	x
LLRS	Impact Status	MSA+	MSA+	MSA+	x	x
	Active Wells	1	1	1	x	x
	Wellpads	1	1	1	x	x
	pH	6.86	6.54	6.75	x	x
LCS	Impact Status	MSA+	MSA+	MSA+	x	x
	Active Wells	2	9	10	x	x
	Wellpads	4	4	4	x	x

	pH	6.4	6.71	6.33	x	x
**Stream**		**2012**	**2013**	**2014**	**2015**	**2016**
LiLRS	Impact Status	MSA+	MSA+	MSA+	MSA+	MSA+
	Active Wells	20	21	21	21	20
	Wellpads	8	8	8	8	8
	pH	4.5	3.98	4.37	4.68	4.64
LWS	Impact Status	MSA+	MSA+	MSA+	MSA+	MSA+
	Active Wells	3	3	3	3	0
	Wellpads	2	2	2	2	2
	pH	7.32	6.28	6.69	6.5	6.94
LoRS	Impact Status	MSA+	MSA+	MSA+	x	MSA+
	Active Wells	0	1	3	x	0
	Wellpads	2	2	2	x	4
	pH	7.48	7.05	7.38	x	7.31
NHS	Impact Status	x	x	MSA+	x	MSA+
	Active Wells	x	x	3	x	0
	Wellpads	x	x	3	x	4
	pH	x	x	7.47	x	7.37
PCS	Impact Status	x	x	MSA+	MSA+	x
	Active Wells	x	x	9	9	x
	Wellpads	x	x	1	1	x
	pH	x	x	4.81	6.1	x
SRCS	Impact Status	MSA+	MSA+	MSA+	x	MSA+
	Active Wells	0	0	1	x	1
	Wellpads	1	1	1	x	1
	pH	7.66	7.02	6.77	x	7.12
SRS	Impact Status	MSA+	MSA+	MSA+	MSA+	x
	Active Wells	13	13	13	13	x
	Wellpads	3	3	3	3	x
	pH	5.01	5.19	5.39	5.42	x
TRTRS	Impact Status	MSA+	MSA+	MSA+	x	x
	Active Wells	9	0	9	x	x
	Wellpads	9	9	9	x	x
	pH	6.99	6.62	6.08	x	x
UTRS	Impact Status	x	x	MSA+	x	x
	Active Wells	x	x	0	x	x
	Wellpads	x	x	0	x	x
	pH	x	x	5.6	x	x

Status: MSA-

Stream		2012	2013	2014	2015	2016
CroRS	Impact Status	MSA-	MSA-	MSA-	MSA-	x
	Active Wells	0	0	0	0	x
	Wellpads	0	0	0	0	x
	pH	5.38	5.41	5.22	5.27	x
DiaRS	Impact Status	MSA-	x	x	x	x
	Active Wells	0	x	x	x	x
	Wellpads	0	x	x	x	x
	pH	7.8	x	x	x	x
DHS	Impact Status	x	x	x	MSA-	MSA-
	Active Wells	x	x	x	0	0
	Wellpads	x	x	x	0	0
	pH	x	x	x	6.67	7.52
FRS	Impact Status	MSA-	MSA-	x	x	x
	Active Wells	0	0	x	x	x
	Wellpads	0	0	x	x	x
	pH	7.78	7.71	x	x	x
MRS	Impact Status	x	x	MSA-	MSA-	MSA-
	Active Wells	x	x	0	0	0
	Wellpads	x	x	0	0	0
	pH	x	x	6.82	6.42	7.2
UTCS	Impact Status	MSA-	MSA-	MSA-	x	MSA-
	Active Wells	0	0	0	x	0
	Wellpads	0	0	0	x	0
	pH	7.9	7.36	7.57	x	7.71
VRS	Impact Status	MSA-	MSA-	MSA-	x	x
	Active Wells	0	0	0	x	x
	Wellpads	0	0	0	x	x
	pH	7.67	7.45	7.12	x	x
WRS	Impact Status	x	MSA-	MSA-	MSA-	x
	Active Wells	x	0	0	0	x
	Wellpads	x	0	0	0	x
	pH	x	5.95	5.94	5.56	x
UBS	Impact Status	x	x	x	MSA-	x
	Active Wells	x	x	x	0	x
	Wellpads	x	x	x	0	x
	pH	x	x	x	6.85	x

Status: MSA- to MSA+

Stream		2012	2013	2014	2015	2016
DRS	Impact Status	MSA-	MSA+	MSA+	MSA+	MSA+
	Active Wells	0	2	1	3	0
	Wellpads	0	2	2	2	2
	pH	5.57	6	5.54	6.73	7.42
UDBS	Impact Status	MSA-	MSA-	MSA+	MSA+	x
	Active Wells	0	0	6	6	x
	Wellpads	0	0	1	1	x
	pH	7.41	7.15	6.85	9.42	x
UEEFW	Impact Status	x	x	MSA-	MSA+	MSA+
	Active Wells	x	x	0	11	0
	Wellpads	x	x	0	1	1
	pH	x	x	6.46	7.3	6.92
UNHS	Impact Status	x	x	MSA-	MSA+	MSA+
	Active Wells	x	x	0	10	0
	Wellpads	x	x	0	1	1
	pH	x	x	5.88	5.13	5.98
UWEFS	Impact Status	x	x	MSA-	MSA+	MSA+
	Active Wells	x	x	0	11	0
	Wellpads	x	x	0	2	2
	pH	x	x	5.38	6.48	6.96

Table S2. Sequence data of all samples (n=189) pre- and post-filtering.

Sample ID	Year	Raw Sequences	Filtered Sequences	OTUs Post Clustering
ALXS_2012	2012	165080	103485	5896
BCM_2012	2012	120858	80648	7823
BCS_2012	2012	45660	28531	5618
BigS_2012	2012	199367	125918	8796
CaRS_2012	2012	384720	248904	15199
CroRS_2012	2012	87051	54651	4505
CSM_2012	2012	84985	54091	4655
CSS_2012	2012	86154	54955	5787
DiaRM_2012	2012	131770	86842	8666
DiaRS_2012	2012	156834	102959	10376
DixRS_2012	2012	114839	71592	6706
DRM_2012	2012	99054	60823	5744
DRS_2012	2012	71673	42940	4358
FRM_2012	2012	32729	21858	4551
FRS_2012	2012	137539	88141	10078
HRM_2012	2012	18811	12203	2797
HRS_2012	2012	90497	58088	8311
LCM_2012	2012	91377	59089	4744
LCS_2012	2012	100621	62064	6238
LiLRS_2012	2012	93698	52845	4104
LLRM_2012	2012	140429	91214	8328
LLRS_2012	2012	46228	30151	5201
LoRM_2012	2012	79935	52933	6195
LoRS_2012	2012	43174	28165	5794
LWM_2012	2012	20396	14051	2228
LWS_2012	2012	109567	69284	7191
SiCM_2012	2012	79277	51111	5340
SRCS_2012	2012	83072	54495	7515
SRM_2012	2012	83405	52092	3356
SRS_2012	2012	86038	53584	3924
STRCS_2012	2012	79404	49926	7553
STRM_2012	2012	10420	6876	1667
ALXS_2013	2013	248541	101783	8941
ALXWL_2013	2013	158292	63300	6955
CaRWL_2013	2013	99742	37975	4797
CSS_2013	2013	323746	146861	11850
CSWL_2013	2013	300086	140136	9198
CroRB_2013	2013	224782	105629	9112
DRM_2013	2013	322732	156753	7394
DRS_2013	2013	277316	127885	8493
DRWL_2013	2013	222016	106121	6869
DixRM_2013	2013	289208	140120	2623

DixRS_2013	2013	265997	123565	9310
FRS_2013	2013	298243	138759	11680
LRM_2013	2013	338313	150849	8660
LRWL_2013	2013	24147	12182	1865
LCB_2013	2013	259249	130672	7594
LCM_2013	2013	335766	160736	10394
LCWL_2013	2013	97512	41126	4449
LWRM_2013	2013	229761	107670	7739
LWRS_2013	2013	297675	139288	10648
LWRWL_2013	2013	59187	22319	1476
LoRM_2013	2013	464117	215811	13079
LoRS_2013	2013	380866	173818	12689
SRCB_2013	2013	261187	131242	7686
SRCM_2013	2013	113172	51664	4557
SRCS_2013	2013	294740	138933	13760
SRS_2013	2013	303912	142932	12149
SRWL_2013	2013	280249	132378	10038
STRCSS_2013	2013	553598	256734	17286
STRCSM_2013	2013	216421	104135	8552
STRCSWL_2013	2013	103929	43576	6868
TRTRM_2013	2013	239597	112721	7953
TRTRS_2013	2013	165287	69511	5537
UTCWL_2013	2013	174640	70757	4465
UDBM_2013	2013	327780	157270	11287
IRM_2013	2013	285382	137552	8712
IRS_2013	2013	501851	224669	11291
VRM_2013	2013	217444	105384	8284
VRS_2013	2013	375121	178725	12272
LiLRS_2013	2013	315239	138210	8192
WRM_2013	2013	595386	265016	13276
WRS_2013	2013	313494	150499	12587
ALXS_2014	2014	208819	152219	10961
CaRS_2014	2014	100619	70467	8662
CaRWL_2014	2014	220379	133637	9589
CroRS_2014	2014	118027	81934	8969
CroRWL_2014	2014	66963	41607	1691
CSS_2014	2014	132955	93976	8840
CSWL_2014	2014	53262	28963	1764
DixRS_2014	2014	229891	169818	9878
DixRWL_2014	2014	65724	36719	2256
DRS_2014	2014	238686	173601	13709
DRWL_2014	2014	115386	66642	2506
IRS_2014	2014	214823	157212	13127
LCS_2014	2014	279060	202885	10868
LCWL_2014	2014	76788	41855	2603

LiLRS_2014	2014	89378	62195	5172
LiLRWL_2014	2014	57187	21187	3374
LoRWL_2014	2014	75027	47411	2197
LRS_2014	2014	160140	114043	13016
LRWL_2014	2014	213533	122428	6989
LWRS_2014	2014	116215	82631	9287
LWRWL_2014	2014	62091	32383	2098
MRS_2014	2014	174258	123526	10996
MRWL_2014	2014	374320	242261	3921
NHWL_2014	2014	46204	20523	3672
PCWL_2014	2014	97389	48365	4298
SiCS_2014	2014	66043	47681	4544
SiCWL_2014	2014	117700	66208	6251
SRCS_2014	2014	117528	79843	8376
SRCWL_2014	2014	116854	55133	5697
SRRSP_2014	2014	292750	176216	9410
SRS_2014	2014	99266	73125	8265
SRWL_2014	2014	109173	50695	2941
STRCSS_2014	2014	196724	138495	13469
STRCSWL_2014	2014	138312	61506	4163
TRTRS_2014	2014	116009	87217	8624
TRTRWL_2014	2014	191428	105151	6435
UDBS_2014	2014	127150	89704	9359
UDBWL_2014	2014	54112	28101	3345
UEEFS_2014	2014	199320	150268	9757
UEEFWL_2014	2014	191016	102972	4748
UNHS_2014	2014	146731	107546	8698
UNHWL_2014	2014	60274	29868	3527
UTCS_2014	2014	113621	83654	10556
UTCWL_2014	2014	98356	54058	4518
UTRWL_2014	2014	64910	26222	2182
UWEFS_2014	2014	169870	123693	12388
UWEFWL_2014	2014	125443	57236	4932
VRS_2014	2014	155631	111587	10072
VRWL_2014	2014	103331	56525	2331
WRS_2014	2014	62986	44355	5893
WRWL_2014	2014	57184	20673	4323
ALXF_2015	2015	98322	58969	1312
ALXS_2015	2015	94206	59889	5332
ALXWL_2015	2015	251591	151189	8698
CroRS_2015	2015	149006	94806	7185
CroRWL_2015	2015	181414	105915	7374
CSS_2015	2015	210457	123034	7247
CSWL_2015	2015	224456	136217	8556
DHS_2015	2015	369808	235881	10546

DHWL_2015	2015	244711	145231	7953
DixRS_2015	2015	313739	195366	10260
DixRWL_2015	2015	188398	101170	5558
DRWL_2015	2015	125285	69038	7930
HRS_2015	2015	354751	220426	12500
HRWL_2015	2015	124288	76257	7616
LiLRB_2015	2015	22162	14709	1641
LiLRS_2015	2015	46778	29829	3550
LiLRWL_2015	2015	174451	86459	3615
LWRF_2015	2015	204866	121758	8248
LWRS_2015	2015	180398	108864	6428
LWRWL_2015	2015	117821	67899	5327
MRS_2015	2015	246323	150344	7811
MRWL_2015	2015	167532	80375	2769
PCS_2015	2015	38542	23656	2955
PCWL_2015	2015	121754	74414	7576
SiCWL_2015	2015	266391	169358	14327
SRS_2015	2015	362760	222217	7821
SRWL_2015	2015	100885	60992	3806
UBS_2015	2015	524429	325239	10281
UBWL_2015	2015	251042	149261	8256
UDBS_2015	2015	153442	97711	5392
UDBWL_2015	2015	195339	119244	9426
UEEFS_2015	2015	534948	325348	9505
UEEFWL_2015	2015	290155	168650	8705
UNHS_2015	2015	563275	335015	6057
UNHWL_2015	2015	255512	163955	5326
UWEFS_2015	2015	316641	199216	11284
UWEFWL_2015	2015	129235	73412	4819
WRS_2015	2015	377016	241699	10768
WRWL_2015	2015	115217	55499	4934
ALXS_2016	2016	494270	262568	10222
ALXWL_2016	2016	50852	26879	4694
CSS_2016	2016	119897	62316	5445
DHS_2016	2016	115827	61737	3695
DixRS_2016	2016	98612	50645	5848
DixRWL_2016	2016	10444	5423	300
DRS_2016	2016	103490	55313	5894
DRWL_2016	2016	27754	14963	504
LiLRS_2016	2016	151063	78954	9561
LiLRWL_2016	2016	114401	60159	6082
LRS_2016	2016	71710	37192	4799
LWS_2016	2016	83695	43375	4143
LWWL_2016	2016	153139	80082	8551
MRS_2016	2016	99087	51691	5488

MRWL_2016	2016	70662	36662	2603
NHS_2016	2016	54668	28851	3662
NHWL_2016	2016	638084	347486	12801
SiCS_2016	2016	70929	37467	5445
SiCWL_2016	2016	141792	73480	9723
SRCS_2016	2016	181965	98581	8052
SRCWL_2016	2016	70835	36787	1669
UEEFS_2016	2016	130334	67766	8607
UNHWL_2016	2016	295830	155094	9672
UTCS_2016	2016	105633	54850	5805
UTCWL_2016	2016	201657	106988	11219
UWEFWL_2016	2016	119427	62449	8135

Table S3. Spearman rank correlations of all measured stream chemistry and watershed characteristics

	**pH**	**TDS**	**Temp**	**Salinity**	**MSA**	**HF**	**Active Wells**	**Wellpads**
**TDS**	rho= 0.38; p=5.99E-05							
**Temp**	rho=-0.18; p=0.063	rho=0.17; p=0.081						
**Salinity**	rho=0.33; p=0.007	rho=0.91; p=2.2E-16	rho=0.40; p=2.27E-05					
**MSA**	rho=-0.32; p=0.008	rho=-0.15; p=0.13	rho=0.03; p=0.79	rho=-0.16; p=0.11				
**HF**	rho=-0.43; p=4.15E-06	rho=-0.07; p=0.47	rho=0.03; p=0.73	rho=-0.13; p=0.18	rho= 0.79; p=2.2E-16			
**Active Wells**	rho=-0.52; p=9.44E-09	rho=-0.13; p=0.17	rho=0.29; p=0.003	rho=-0.07; p=0.49	rho=0.58; p=7.62E-11	rho=0.70; p=2.2E-16		
**Wellpads**	rho=-0.43; p=4.24E-06	rho=-0.08; p=0.40	rho=0.18; p=0.07	rho=-0.08; p=0.44	rho=0.78; p=2.2E-16	rho=0.75; p=2.2E-16	rho=0.65; p=8.28E-14	
**Year**	rho=-0.19; p=0.06	rho=-0.19; p=0.05	rho=-0.36; p=0.001	rho=-0.25; p=0.01	rho=0.09; p=0.34	rho=0.14; p=0.16	rho=0.07; p=0.49	rho=0.06; p=0.51

Table S4. Analysis of Similarity (ANOSIM) p-values for each respective year between MSA+ and MSA- categorized streams.

Sampling Year	ANOSIM P-values
**2012**	0.174
**2013**	0.132
**2014**	0.515
**2015**	0.22
**2016**	0.505

Table S5. Alpha diversity measure for sediment, water, and biofilm samples over 5 years

Sample Matrix	Group	Heip's evenness	Observed Species	Chao1
Sediment	MSA+	0.3523 ± 0.0779	2738.76 ± 499.72	5561.29 ± 1307.11
	MSA-	0.3834 ± 0.0799	2898.05 ± 444.29	5901.58 ± 1225.55
Water	MSA+	0.2736 ± 0.1822	1071.65 ± 371.51	1367.15 ± 424.09
	MSA-	0.2260 ± 0.1879	1050.28 ± 374.52	1364.42 ± 396.56
Biofilm	MSA+	0.1552 ± 0.0585	808.76 ± 167.48	1098.16 ± 201.35
	MSA-	0.2364	1155.5	1611.10

Table S6. Mean Decrease in Gini Index table from Random Forest modelling

OTU_ID		GINI AVG
**OTU_58872**	Sphingobacteriales	0.143075631
**OTU_3292**	Cytophagaceae	0.110412712
**OTU_712**	Solibacterales	0.094517591
**OTU_14130**	Betaproteobacteria	0.083141135
**OTU_66510**	Stramenopiles	0.078261448
**OTU_999**	[Bryobacteraceae]	0.075362258
**OTU_62597**	Acetobacteraceae	0.073725944
**OTU_44095**	Hyphomicrobiaceae	0.072964316
**OTU_616**	Nitrospira	0.069665763
**OTU_13242**	Betaproteobacteria	0.066812412
**OTU_941**	Isosphaeraceae	0.066530169
**OTU_1565**	Chloroflexi	0.065158955
**OTU_471**	Myxococcales	0.064608405
**OTU_395**	Myxococcales	0.063770943
**OTU_42122**	Alphaproteobacteria	0.062890431
**OTU_41164**	Acidobacteriaceae	0.061235873
**OTU_351**	Fcolumnare	0.060854773
**OTU_28029**	EB1017	0.060834246
**OTU_45226**	Steroidobacter	0.060782813
**OTU_44555**	Acidimicrobiales	0.057866196
**OTU_605**	PBS-25	0.057200679
**OTU_768**	C111	0.056849844
**OTU_55965**	Proteobacteria	0.056110856
**OTU_58141**	Acidobacteriaceae	0.056104947
**OTU_396**	Rhizobiales	0.05583198
**OTU_23247**	Comamonadaceae	0.0546884
**OTU_50**	Phenylobacterium	0.052699116
**OTU_757**	Betaproteobacteria	0.052009611
**OTU_841**	Ellin515	0.051786588
**OTU_54504**	Betaproteobacteria	0.051556483

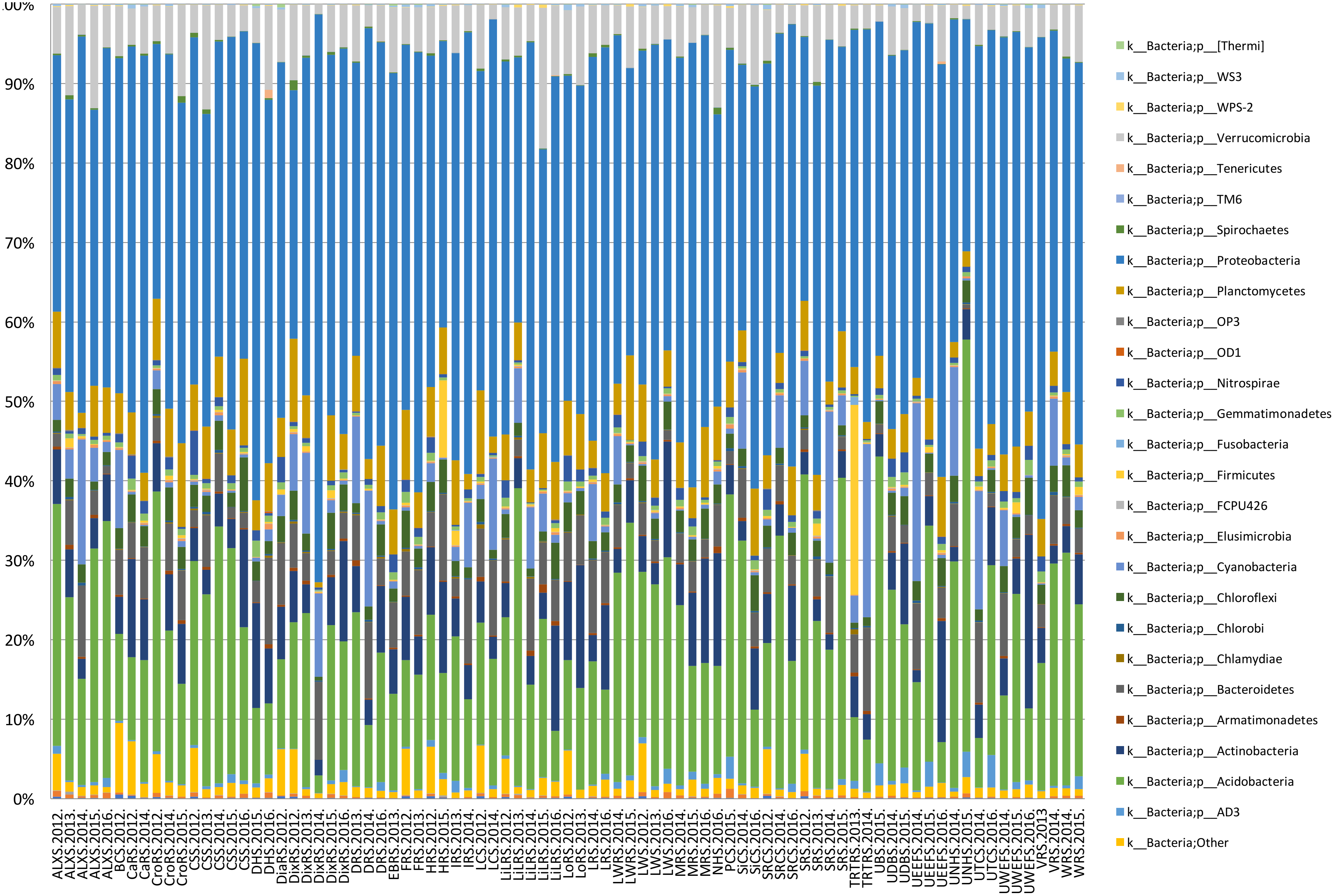

Figure S1. Phylum level abundance of sediment samples across all years. Abundance plot was generated with an unrarefied OTU table in Excel, filtered the Phylum level. Samples are displayed along the x-axis and relative abundance in percentage is displayed along the y-axis.

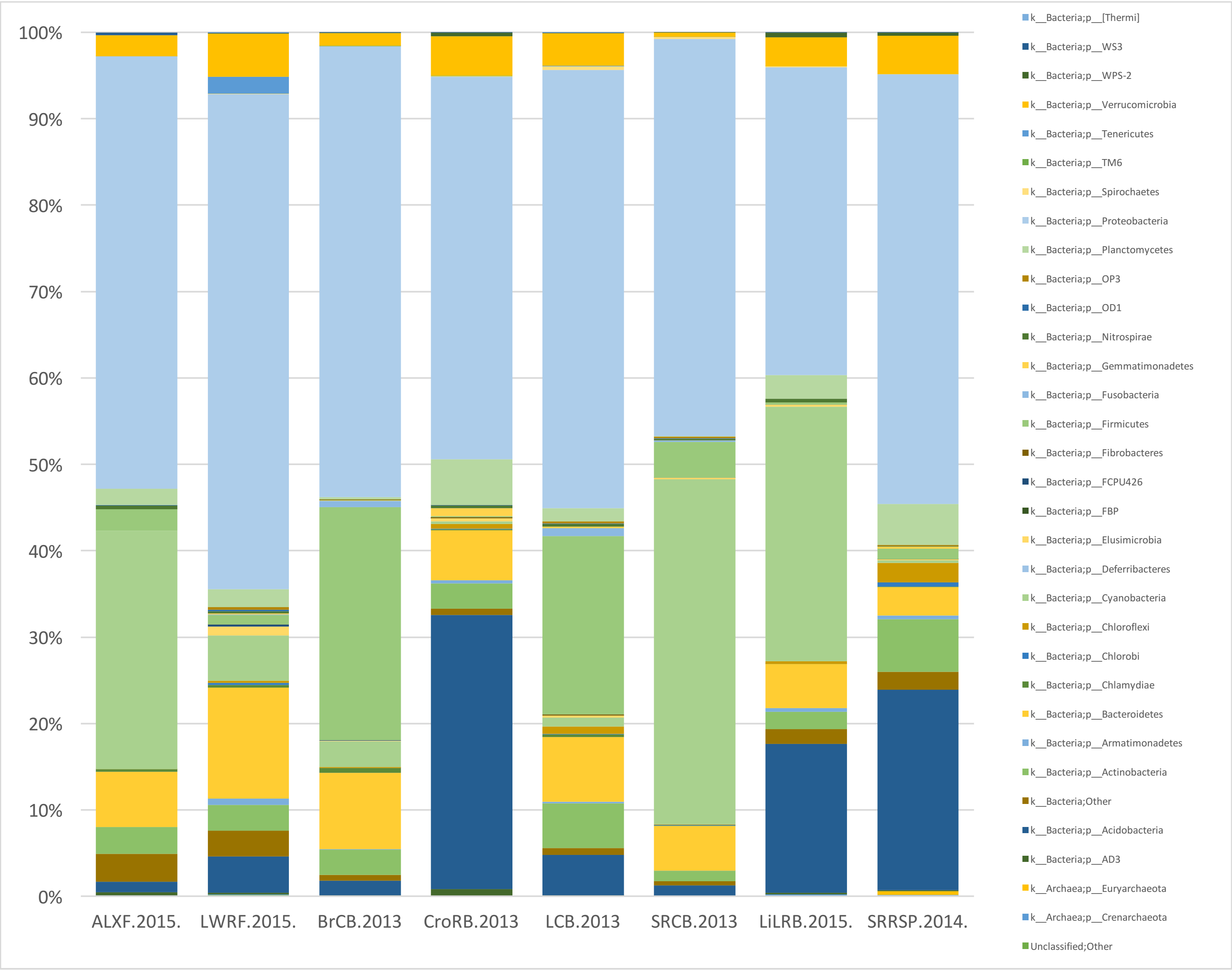

Figure S2. Biofilm, seepage, and foam phylum-level diversity. Abundance plot was generated with an unrarefied OTU table in Excel, filtered the Phylum level. Samples are displayed along the x-axis and relative abundance in percentage is displayed along the y-axis.

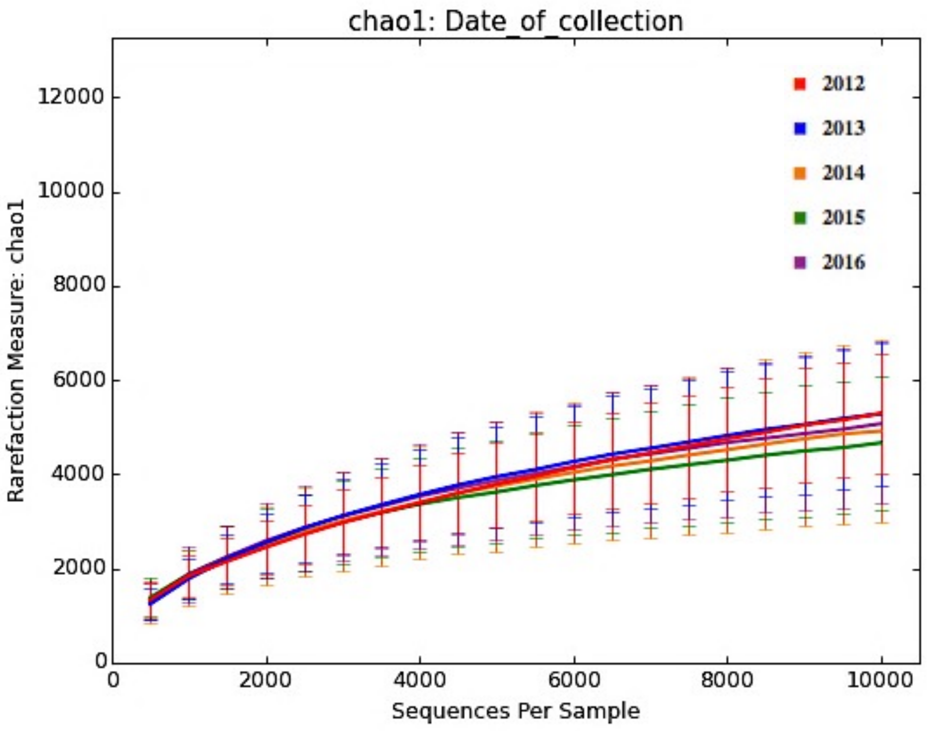

Figure S3. Rarefaction curves of each year (2012-2016) generated using QIIME 1.9.0. Multiple rarefactions were conducted on across all samples from minimum depth of 500 sequences, to a maximum depth of 10,000 sequences, with a step size of 500 sequences/sample for 10 iterations. Alpha rarefactions were then collated and plotted using Chao1 and observed species richness metrics. Rarefaction curves present sequencing depth on the x-axis, and the number of unique species on the y-axis.

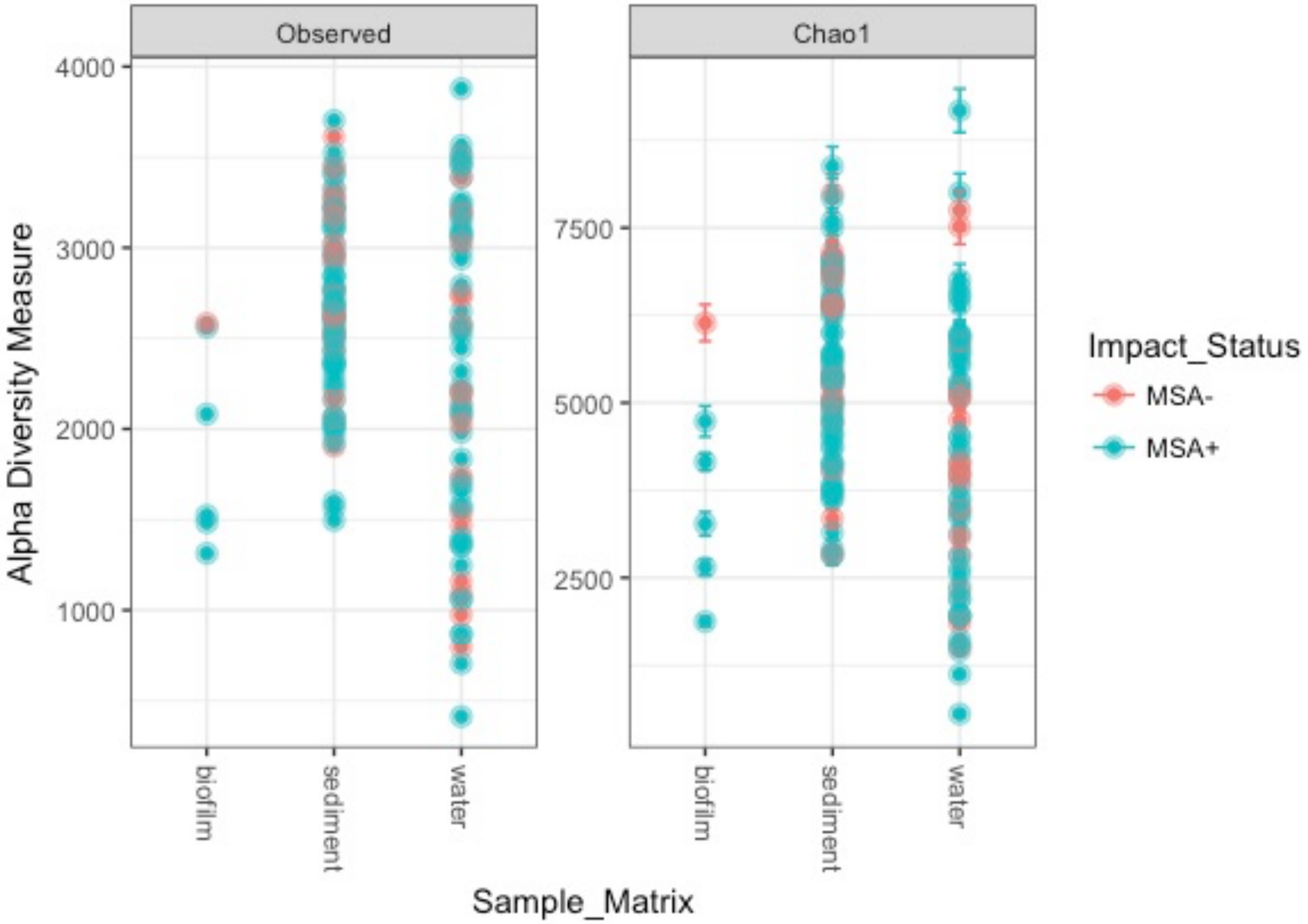

Figure S4. Alpha diversity of sediment, water, and biofilm samples. Species richness was estimated by performing multiple rarefactions up to a depth of 10,000 sequences per sample. The richness of OTUs from the maximum rarefaction depth was calculated using observed richness and Chao1 and visualized using phyloseq in R. For each metric, species richness is colored by Marcellus Shale Activity: MSA+ as blue and MSA- as red.

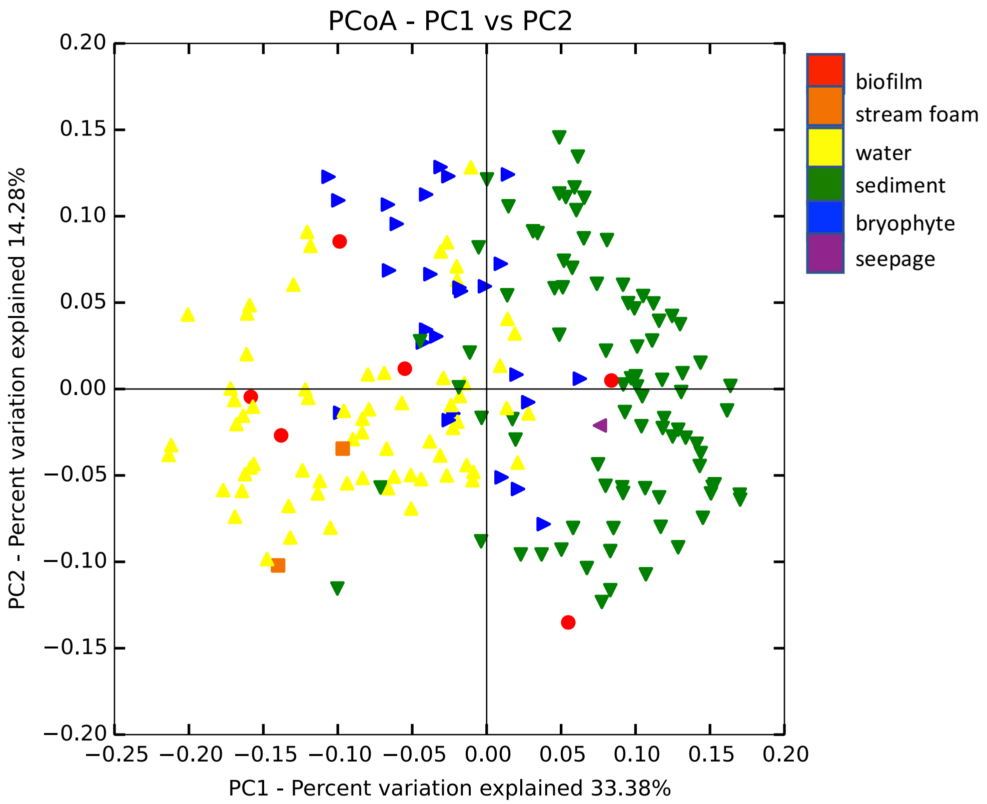

Figure S5. Beta diversity of stream bacteria communities based on sample matrix. Principal-coordinate analysis (PCoA) plot generated using weighted Unifrac distances of all samples from a cumulative sum scaling (CSS)-normalized OTU table. Sediment samples are green; water samples are yellow; bryophyte samples are blue; biofilm samples are red; seepage sample is purple; foam samples are orange.

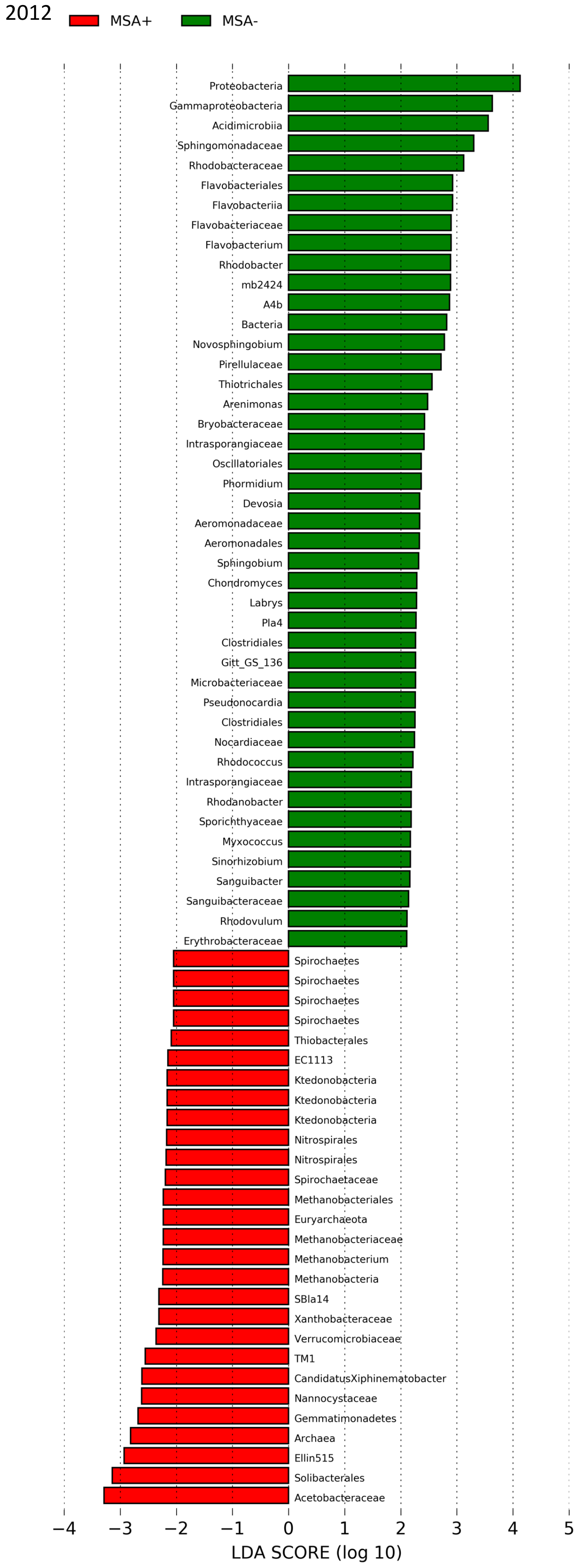
2012
MSA+
MSA-
Proteobacteria
Gammaproteobacteria
Acidimicrobiia
Sphingomonadaceae
Rhodobacteraceae
Flavobacteriales
Flavobacteriia
Flavobacteriaceae
Flavobacterium
Rhodobacter
mb2424
A4b
Bacteria
Novosphingobium
Pirellulaceae
Thiotrichales
Arenimonas
Bryobacteraceae
Intrasporangiaceae
Oscillatoriales
Phormidium
Devosia
Aeromonadaceae
Aeromonadales
Sphingobium
Chondromyces
Labrys
Pla4
Clostridiales
Gitt_GS_136
Microbacteriaceae
Pseudonocardia
Clostridiales
Nocardiaceae
Rhodococcus
Intrasporangiaceae
Rhodanobacter
Sporichthyaceae
Myxococcus
Sinorhizobium
Sanguibacter
Sanguibacteraceae
Rhodovulum
Erythrobacteraceae
Spirochaetes
Spirochaetes
Spirochaetes
Spirochaetes
Thiobacterales
EC1113
Ktedonobacteria
Ktedonobacteria
Ktedonobacteria
Nitrospirales
Nitrospirales
Spirochaetaceae
Methanobacteriales
Euryarchaeota
Methanobacteriaceae
Methanobacterium
Methanobacteria
SBla14
Xanthobacteraceae
Verrucomicrobiaceae
TM1
CandidatusXiphinematobacter
Nannocystaceae
Gemmatimonadetes
Archaea
Ellin515
Solibacterales
Acetobacteraceae
−4
−3
−2
−1
0
1
2
3
4
5
LDA SCORE (log 10)

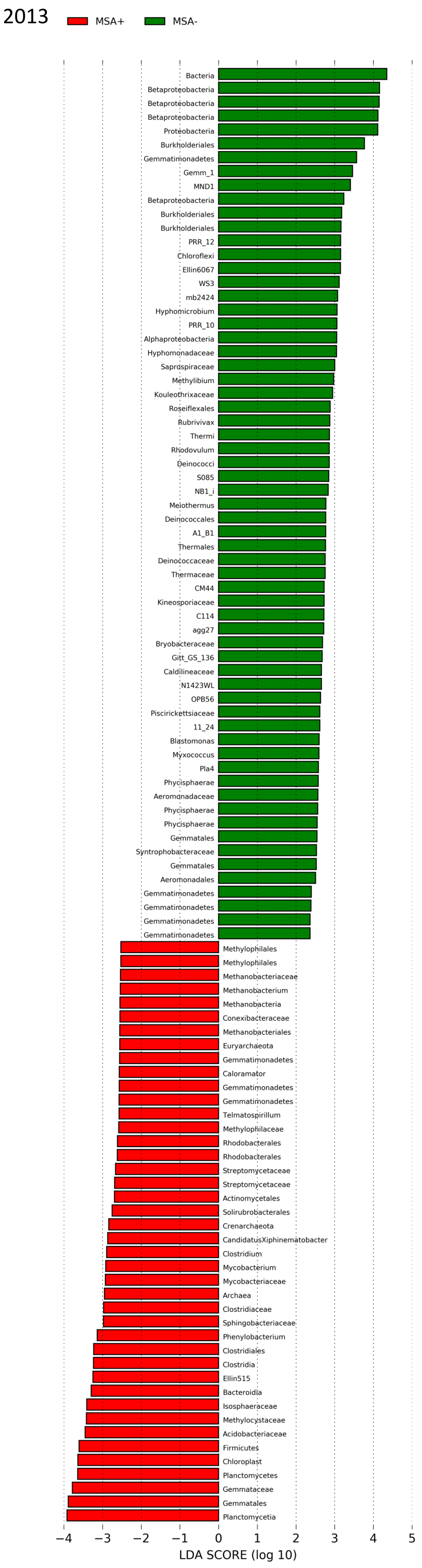
2013
MSA+
MSA-
Bacteria
Betaproteobacteria
Betaproteobacteria
Betaproteobacteria
Proteobacteria
Burkholderiales
Gemmatimonadetes
Gemm_1
MND1
Betaproteobacteria
Burkholderiales
Burkholderiales
PRR_12
Chloroflexi
Ellin6067
WS3
mb2424
Hyphomicrobium
PRR_10
Alphaproteobacteria
Hyphomonadaceae
Saprospiraceae
Methylibium
Kouleothrixaceae
Roseiflexales
Rubrivivax
Thermi
Rhodovulum
Deinococci
S085
NB1_i
Meiothermus
Deinococcales
A1_B1
Thermales
Deinococcaceae
Thermaceae
CM44
Kineosporiaceae
C114
agg27
Bryobacteraceae
Gitt_GS_136
Caldilineaceae
N1423WL
OPB56
Piscirickettsiaceae
11_24
Blastomonas
Myxococcus
Pla4
Phycisphaerae
Aeromonadaceae
Phycisphaerae
Phycisphaerae
Gemmatales
Syntrophobacteraceae
Gemmatales
Aeromonadales
Gemmatimonadetes
Gemmatimonadetes
Gemmatimonadetes
Gemmatimonadetes
Methylophilales
Methylophilales
Methanobacteriaceae
Methanobacterium
Methanobacteria
Conexibacteraceae
Methanobacteriales
Euryarchaeota
Gemmatimonadetes
Caloramator
Gemmatimonadetes
Gemmatimonadetes
Telmatospirillum
Methylophilaceae
Rhodobacterales
Rhodobacterales
Streptomycetaceae
Streptomycetaceae
Actinomycetales
Solirubrobacterales
Crenarchaeota
CandidatusXiphinematobacter
Clostridium
Mycobacterium
Mycobacteriaceae
Archaea
Clostridiaceae
Sphingobacteriaceae
Phenylobacterium
Clostridiales
Clostridia
Ellin515
Bacteroidia
Isosphaeraceae
Methylocystaceae
Acidobacteriaceae
Firmicutes
Chloroplast
Planctomycetes
Gemmataceae
Gemmatales
Planctomycetia
−4
−3
−2
−1
0
1
2
3
4
5
LDA SCORE (log 10)

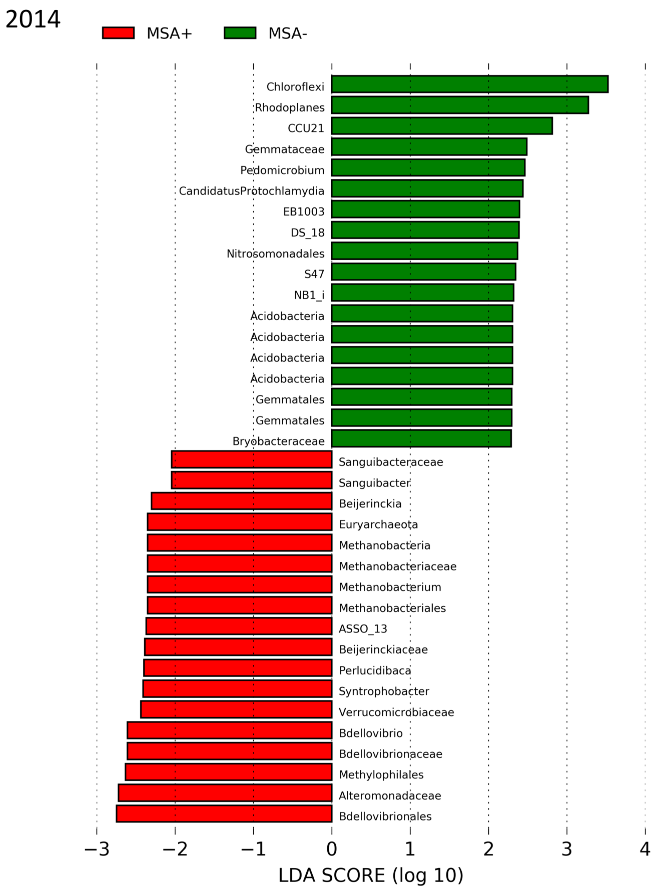

2014
MSA+
MSA-
Chloroflexi
Rhodoplanes
CCU21
Gemmataceae
Pedomicrobium
CandidatusProtochlamydia
EB1003
DS_18
Nitrosomonadales
S47
NB1_i
Acidobacteria
Acidobacteria
Acidobacteria
Acidobacteria
Gemmatales
Gemmatales
Bryobacteraceae
Sanguibacteraceae
Sanguibacter
Beijerinckia
Euryarchaeota
Methanobacteria
Methanobacteriaceae
Methanobacterium
Methanobacteriales
ASSO_13
Beijerinckiaceae
Perlucidibaca
Syntrophobacter
Verrucomicrobiaceae
Bdellovibrio
Bdellovibrionaceae
Methylophilales
Alteromonadaceae
Bdellovibrionales
−3
−2
−1
0
1
2
3
4
LDA SCORE (log 10)

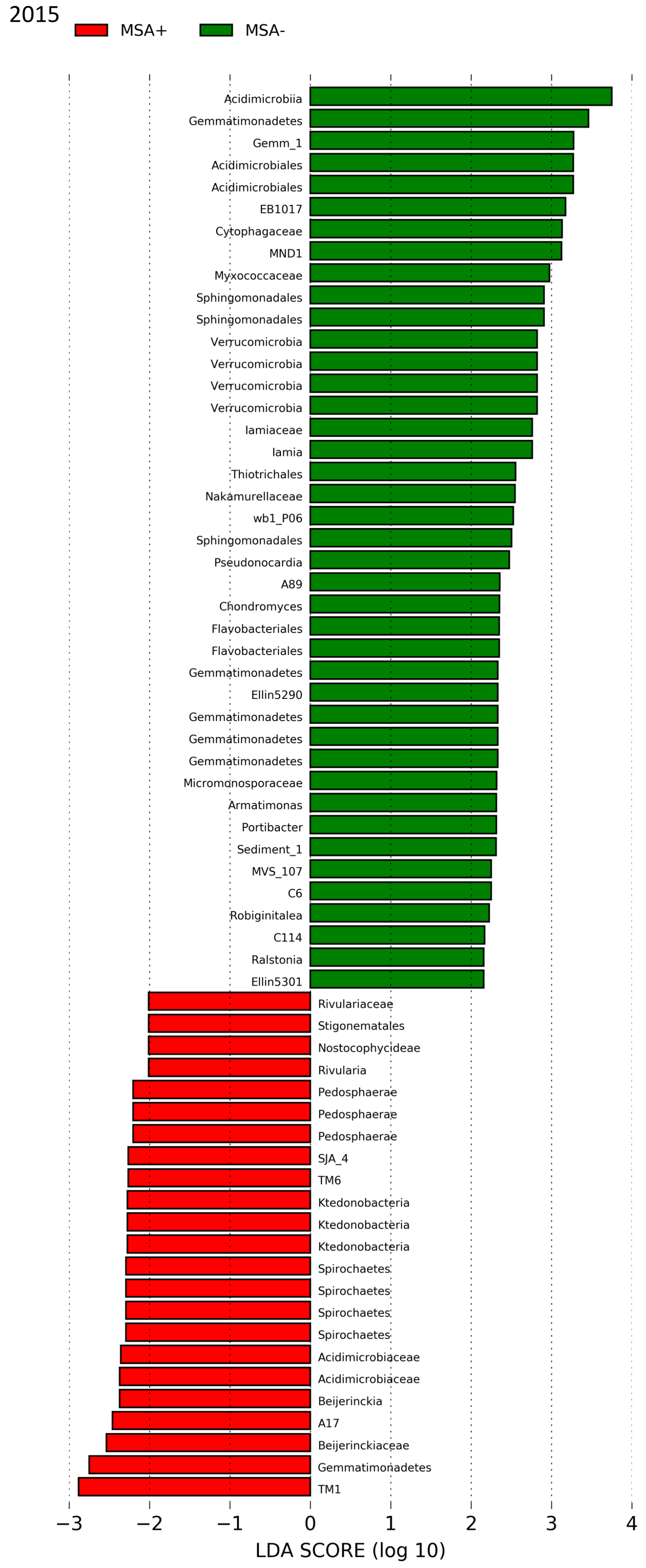
2015
MSA+
MSA-
Acidimicrobiia
Gemmatimonadetes
Gemm_1
Acidimicrobiales
Acidimicrobiales
EB1017
Cytophagaceae
MND1
Myxococcaceae
Sphingomonadales
Sphingomonadales
Verrucomicrobia
Verrucomicrobia
Verrucomicrobia
Verrucomicrobia
Iamiaceae
Iamia
Thiotrichales
Nakamurellaceae
wb1_P06
Sphingomonadales
Pseudonocardia
A89
Chondromyces
Flavobacteriales
Flavobacteriales
Gemmatimonadetes
Ellin5290
Gemmatimonadetes
Gemmatimonadetes
Gemmatimonadetes
Micromonosporaceae
Armatimonas
Portibacter
Sediment_1
MVS_107
C6
Robiginitalea
C114
Ralstonia
Ellin5301
Rivulariaceae
Stigonematales
Nostocophycideae
Rivularia
Pedosphaerae
Pedosphaerae
Pedosphaerae
SJA_4
TM6
Ktedonobacteria
Ktedonobacteria
Ktedonobacteria
Spirochaetes
Spirochaetes
Spirochaetes
Spirochaetes
Acidimicrobiaceae
Acidimicrobiaceae
Beijerinckia
A17
Beijerinckiaceae
Gemmatimonadetes
TM1
−3
−2
−1
0
1
2
3
4
LDA SCORE (log 10)

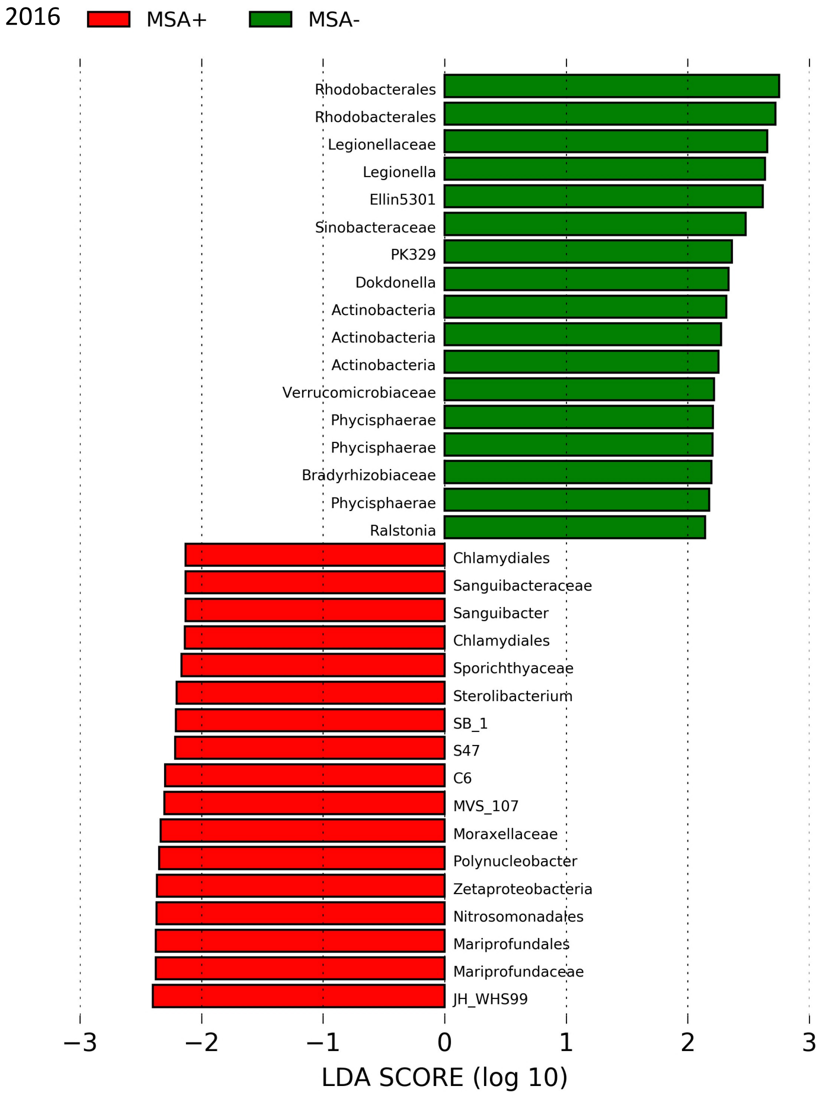

Figure S6. Multiple-page LEfSe plots of taxonomic “biomarkers” MSA+ and MSA- communities separated by sample year 2012-2016 of sediment samples. MSA+ = red; MSA- = green. LEfSe utilizes Kruskal-Wallis to determine significantly different taxonomic features ($p < 0.01$) between experimental groups, a pairwise Wilcoxon rank sum statistic to test biological consistency across subgroups ($p < 0.01$), and finally a linear discriminant analysis (LDA score > 2.0) to determine the effect size, or magnitude of variation of the features between groups.
